# Structural Evolution of Iron-Loaded Metal–Organic Framework Catalysts for Continuous Gas-Phase Oxidation of Methane to Methanol

**DOI:** 10.1021/acsami.3c03310

**Published:** 2023-05-23

**Authors:** Bunyarat Rungtaweevoranit, Ali M. Abdel-Mageed, Pongtanawat Khemthong, Srisin Eaimsumang, Khetpakorn Chakarawet, Teera Butburee, Benny Kunkel, Sebastian Wohlrab, Kittipong Chainok, Jakkapop Phanthasri, Suttipong Wannapaiboon, Saran Youngjan, Theerada Seehamongkol, Sarawoot Impeng, Kajornsak Faungnawakij

**Affiliations:** † National Nanotechnology Center (NANOTEC), National Science and Technology Development Agency (NSTDA), Pathum Thani 12120, Thailand; ‡ Leibniz-Institut für Katalyse e.V. (LIKAT Rostock), Albert-Einstein-Straße 29a, 18059 Rostock, Germany; § Department of Chemistry, Faculty of Science, Cairo University, 12613 Giza, Egypt; ∥ Institute of Surface Chemistry and Catalysis, Ulm University, D-89069 Ulm, Germany; ⊥ Department of Chemistry, University of California, Berkeley, Berkeley, California 94720, United States; # Department of Chemistry, Faculty of Science, Mahidol University, Bangkok 10400, Thailand; ∇ Thammasat University Research Unit in Multifunctional Crystalline Materials and Applications (TU-MCMA), Faculty of Science and Technology, Thammasat University, Pathum Thani 12121, Thailand; ○ Synchrotron Light Research Institute (Public Organization), 111 University Avenue, Suranaree, Muang, Nakhon Ratchasima 30000, Thailand

**Keywords:** partial methane oxidation, methanol, metal−organic framework, heterogeneous catalysis, iron catalyst

## Abstract

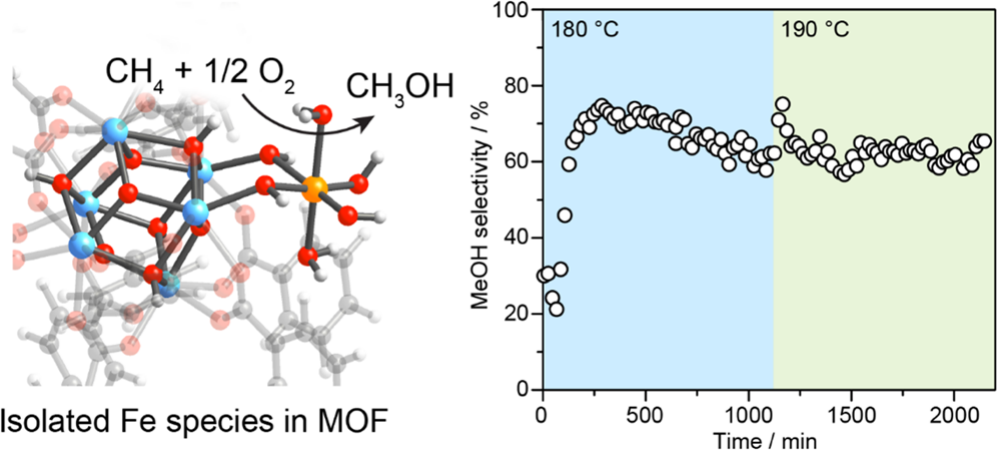

Catalytic partial oxidation of methane presents a promising route to convert the abundant but environmentally undesired methane gas to liquid methanol with applications as an energy carrier and a platform chemical. However, an outstanding challenge for this process remains in developing a catalyst that can oxidize methane selectively to methanol with good activity under continuous flow conditions in the gas phase using O_2_ as an oxidant. Here, we report a Fe catalyst supported by a metal–organic framework (MOF), Fe/UiO-66, for the selective and on-stream partial oxidation of methane to methanol. Kinetic studies indicate the continuous production of methanol at a superior reaction rate of 5.9 × 10^–2^ μmol_MeOH_ g_Fe_^–1^ s^–1^ at 180 °C and high selectivity toward methanol, with the catalytic turnover verified by transient methane isotopic measurements. Through an array of spectroscopic characterizations, electron-deficient Fe species rendered by the MOF support is identified as the probable active site for the reaction.

## 1 Introduction

Partial oxidation of methane to methanol remains a key challenge in catalysis.^1−5^ The intrinsic difficulty stems from (i) the extremely high C–H bond dissociation energy of methane (413 kJ mol^–1^) compared to other alkanes, which requires strong oxidizing catalysts and harsh conditions^2^, (ii) low polarizability, and (iii) the higher reactivity of the methanol product compared to its substrate which is susceptible to further oxidation.^4,6,7^ This inadvertently results in extensive oxidation of all C–H bonds to produce CO_2_ unless a certain process is in place to prevent the subsequent oxidation of the reaction product.^8^ Addressing this impediment could divert the flaring process of highly potent greenhouse gas such as methane to the production of high-valued methanol.^7,9^ Research efforts in this area have largely focused on developing new catalysts and optimizing the reaction conditions for the looping process where the catalysts are exposed sequentially to an oxidant, methane, and water vapor at different reaction temperatures and pressures.^9−15^ While the looping process has been successfully proven in preventing the overoxidation of the methanol product by avoiding the co-presence of an oxidant and methanol, the maximum theoretical turnover number per one cycle is limited to one.^4^ A more desirable option for industrial implementation is a continuous flow process in the gas phase where the reaction proceeds under isothermal conditions without the need of alternating reactant feeds. Thus, the greater challenge remains in developing a catalyst that can selectively oxidize methane to methanol under continuous flow of methane and oxidant, which provides more practical operation and productivity.^16^ Achieving this on-stream process brings us a step forward toward the possible commercialization of direct conversion of methane to methanol.

To date, only Cu-exchanged zeolites are reportedly capable of oxidizing methane to methanol with good selectivity under continuous conditions.^17−20^ Inspired by the soluble methane monooxygenase (sMMO) enzyme which utilizes iron-oxo species to transform methane to methanol under mild conditions,^21^ we envisaged that a synthetic Fe complex could function as an active site for this reaction. Within this context, Fe-exchanged zeolites have been shown as competent catalysts for methane oxidation under looping conditions^22−25^ while isolated Fe sites in MIL-53 and zeolites are reportedly capable of methane oxidation using H_2_O_2_ as an oxidant.^26−28^ However, none of them has been proven to work actively under continuous conditions using O_2_ as an oxidant. This shortcoming is likely associated with the unsuitable electronic properties of the Fe sites.

Here, we report a Fe-based catalyst, comprising Fe sites supported on a Zr-based MOF, UiO-66,^29^ hereafter referred to as Fe/UiO-66, capable of activating methane to methanol under continuous flow conditions in the gas phase using molecular oxygen and water vapor. The catalyst exhibits a stable activity of 5.9 × 10^–2^ μmol_MeOH_ g_Fe_^–1^ s^–1^ and selectivity of 62%. We made use of the well-defined molecular structure of the catalyst to further elucidate the probable active site in this catalyst by employing a combination of ^57^Fe Mössbauer spectroscopy, electron paramagnetic resonance (EPR) spectroscopy, Fe K-edge X-ray absorption spectroscopy (XAS), in situ time-resolved diffuse reflectance UV–vis spectroscopy, and in situ diffuse reflectance infrared Fourier transform spectroscopy (DRIFTS) coupled with density functional theory (DFT) calculations. Electron-deficient and isolated Fe sites coordinated to oxygen ligands residing in the MOF were identified as the active sites for methanol formation.

## 2 Experimental Section

### 2.1 Synthesis of Fe/UiO-66 Catalyst

UiO-66 (600 mg) was added to a solution containing FeCl_3_·6H_2_O (856 mg) dissolved in DMF (9 mL). The suspension was sonicated for 1 min. The vial’s thread was wrapped with PTFE tape, sealed, and heated in an 85 °C isothermal oven for 15 h. The yellow-orange product was collected by centrifugation (10 000 rpm, 5 min) and washed with DMF five times (25 mL × 5) over 3 days and acetone three times (25 mL × 3) over 24 h. The sample was dried under dynamic vacuum overnight at room temperature.

### 2.2 Characterizations

Powder X-ray diffraction patterns (PXRD) were recorded using a Bruker D8 Advance diffractometer (Bragg–Brentano, monochromated Cu Kα radiation λ = 1.54056 Å). N_2_ adsorption isotherms were collected on a Quantachrome iQ-MP/XR volumetric gas adsorption analyzer. A liquid nitrogen bath (77 K) and ultrahigh-purity grade N_2_ and He (99.999%, Praxair) were used for the measurements. Scanning electron microscopic (SEM) images were obtained using a Hitachi SU8030 scanning electron microscope. Transmission electron microscopic (TEM) images were acquired on a Jeol JEM-2100Plus. Metal loadings were measured using inductively coupled plasma optical emission spectroscopic (ICP-OES) analyses using a Shimadzu ICPE-9820. Zero-field ^57^Fe Mössbauer spectra were recorded in a constant acceleration spectrometer (SEE Co., Minneapolis, MN) which utilized a cobalt-57 gamma source embedded in a Rh matrix. Isomer shifts are reported relative to α-iron (30 μm foil) at 295 K. Fe K-edge X-ray absorption spectroscopy data were collected at the BL 5.2 SUT-NANOTEC-SLRI, a bending-magnet beamline with the storage ring operating at a current of 80–150 mA and 1.2 GeV at the Synchrotron Light Research Institute (SLRI), Thailand. In situ infrared spectroscopy was carried out using a Nicolet iS50 equipped with an MCT detector and a high-temperature reaction cell. More details are given in the Supporting Information.

### 2.3 Catalysis

Activity and selectivity measurements were performed at 5 bar in a glass-lined stainless-steel tubular flow microreactor at 180 and 190 °C temperature. A catalyst bed made of 170 mg of pure Fe/UiO-66 catalyst powder sieved to 100–250 μm was delimited in the middle of the microreactor by two plugs of the quartz wool on the top and bottom sides, resulting in a catalyst bed length of about 1.8 cm. Considering a total flow rate of 30 mL min^–1^ and an inner diameter of the microreactor of 0.45 cm, the gas space hourly velocity is 6285 h^–1^. Prior to the kinetic measurements, the catalyst was pretreated according to the following optimized procedure. The catalyst was first activated by heating up from room temperature to 250 °C under a continuous flow of Ar to remove volatile adsorbates, followed by exposure to 10% O_2_/Ar at 250 °C for 1 h, Ar for 15 min, and humidified Ar carrying 5% of water vapor for a period of 1 h at 250 °C, 1 bar. Afterward, the catalyst was dried in Ar while decreasing the temperature from 250 °C to the reaction temperature of 180 °C. Measurements were carried out under differential reaction conditions (methane conversion <0.1%) in an optimized feed gas (10% CH_4_, 5% O_2_ + 0.2% H_2_O, balance Ar). The desired amount of water vapor (0.2%) was introduced during reaction at 5 bar using a high-performance liquid chromatography pump (Jasco PU-980 model) integrated with a custom-made evaporator as described in Figure S9. A backpressure regulator (Tescom ER5000) was applied to control the reaction pressures. Reaction gases were analyzed with a gas chromatograph equipped with TCD detectors and employing a two-stage temperature program for separating methanol from other products (Figure S10). Product distribution was qualitatively cross-checked with online mass spectrometry. To calibrate the retention time of reaction products and their concentration, we employed two different test gas mixtures including mix 1 (1% CO, 1% CO_2_, 1% CH_4_, 1% O_2_, and N_2_ balance) and mix 2 (0.5% methanol in Ar).

Based on the molar flow rate of MeOH (*n*_MeOH_,_out_) or CO_2_ (*n*_CO_2_,out_) and the weight of Fe metal (*m*_Fe_), the Fe mass-normalized MeOH and CO_2_ formation rates (*R*_MeOH_ and *R*_CO_2__) were calculated according to eqs 1 and 2. Based on the Fe-based reaction rates, the atomic mass of Fe (55.85 g mol^–1^), and the Fe dispersion (*D*_Fe_) of 46%, turnover frequencies (TOFs) were calculated according to eq 3. Considering the detection of only methanol and CO_2_, the selectivity for MeOH formation (*S*_MeOH_) is defined as the ratio of the MeOH formation rate to the sum of MeOH and CO_2_, see eq 4.
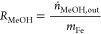
1
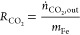
2
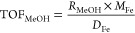
3
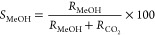
4

### 2.4 In Situ Diffuse Reflectance UV–Vis Spectroscopy (DR UV–Vis)

In situ DR UV–vis was performed on an Ava Spec-2048 spectrometer equipped with an FCR-7UV400C-2 reflection probe (Avantes, Apeldoorn, The Netherlands) in the energy range 1.5–7 eV. The samples were placed in a commercial in situ reaction chamber (HVC-MRA-5, Harrick, Pleasantville, NY). More details are given in the Supporting Information.

## 3 Results and Discussion

### 3.1 Catalyst Synthesis

**Figure 1:**
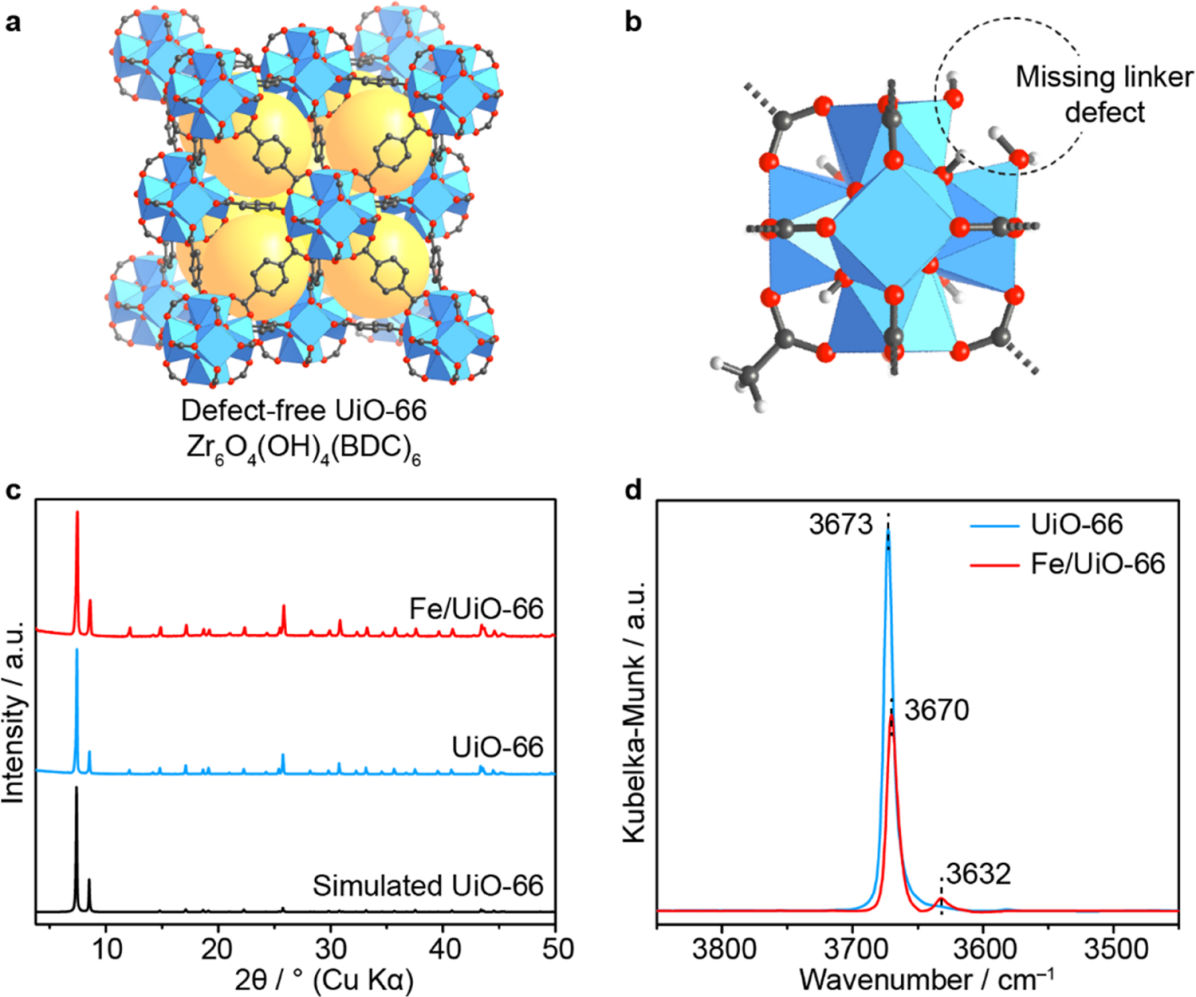
Preparation and characterizations of Fe/UiO-66. (a) Crystal structure of pristine UiO-66 in the absence of the defect sites. Yellow spheres represent the space in the framework. (b) Zr_6_ cluster of UiO-66 containing missing a terephthalate linker defect where these sites are terminated by acetate, OH^–^, and H_2_O molecules. Atom labeling scheme: C, black; O, red; Zr, blue; H, white. (c) Powder X-ray diffraction patterns of UiO-66 and Fe/UiO-66 in comparison with the simulated pattern of UiO-66. (d) In situ DRIFTS spectra of UiO-66 and Fe/UiO-66 measured under the N_2_ atmosphere at 175 °C.

UiO-66 MOF was synthesized by a solvothermal reaction of ZrCl_4_ and terephthalic acid in *N*,*N*-dimethylformamide at 120 °C using acetic acid as a modulator to produce UiO-66 as a microcrystalline solid (Section S1).^30^ This MOF was selected for its hydrolytic stability to resist structural degradation from water vapor during partial methane oxidation.^31,32^ From characterizations and data analysis, the synthesized MOF possesses a chemical formula of Zr_6_O_4_(OH)_4_ (C_8_H_4_O_4_)_5_(CH_3_COO)_0.9_ (OH)_1.2_(H_2_O)_1.2_ (Figures S1, S3, and S4).^33^ Thus, each Zr_6_ node in the framework contains ∼1 acetate molecule and one pair of OH^–^ and H_2_O molecules per Zr_6_ oxide cluster capping the linker missing defect site (Figure 1a,b).^34^ We utilized these defect sites to anchor Fe atoms by heating UiO-66 in a solution of FeCl_3_·6H_2_O in DMF at 85 °C overnight to yield Fe/UiO-66 as orange-brown powder. The powder X-ray diffraction (PXRD) analysis of the as-prepared Fe/UiO-66 sample shows that the resulting material retains its crystallinity with a similar diffraction pattern to that of the pristine UiO-66 indicating that the catalyst adopts the same framework as UiO-66 (Figure 1c). Inductively coupled plasma optical emission spectroscopy (ICP-OES) of Fe/UiO-66 shows the presence of Fe in the catalyst sample with a Fe/Zr_6_ atomic ratio of 1.32 (equivalent to a total wt % of 3.1) which coincides with the amount of the missing linker defect.^33^ The porosity of the catalyst was analyzed by N_2_ sorption isotherm measurement at 77 K showing expectedly diminished BET surface area from 1326 m^2^ g^–1^ in UiO-66 to 1010 m^2^ g^–1^ in Fe/UiO-66 (Figure S3).

To determine the location of the Fe sites, we performed in situ diffuse reflectance FTIR spectroscopy (DRIFTS) on Fe/UiO-66 and UiO-66 under a nitrogen atmosphere at 175 °C to avoid the spectral interference from physisorbed water molecules (Figure 1d). The intensity of the O–H related bands located at 3673 cm^–1^ decreases along with a slight red shift to 3670 cm^–1^ upon metalation indicating the lower dipole–dipole concentration at lower coverage of the OH^–^/H_2_O pair. This result suggests that Fe is covalently bound to the OH^–^/H_2_O groups terminating the defect sites of UiO-66 by replacing the proton of the H_2_O group.^33,35^ At the same time, a peak at 3632 cm^–1^ appeared in Fe/UiO-66 catalyst which is likely associated with the O–H stretch of hydroxide or water ligands bound to the newly installed Fe site. Additionally, we synthesized single crystals of UiO-66 which were then subjected to the same conditions for the synthesis of Fe/UiO-66. Single-crystal X-ray diffraction (SXRD) analysis of Fe/UiO-66 reveals the excess electron density near the OH^–^/H_2_O site that could be related to the Fe site (Figures S7 and S8). However, further refinement to locate the Fe atoms is proved challenging because of the structural disorder and the interference from guest solvent molecules.

**Figure 2:**
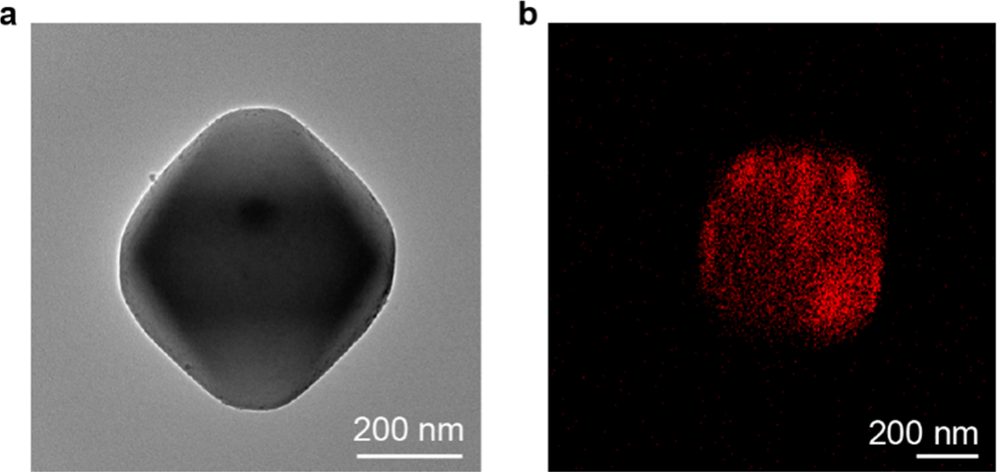
Morphological characterizations of as-synthesized Fe/UiO-66. (a) Transmission electron microscopic image and (b) the corresponding EDX map of the Fe Kα line.

Transmission electron microscopy (TEM) and electron dispersive X-ray spectroscopy (EDX) analyses of the catalyst reveal the presence of Fe distributed inside the MOF crystals (Figure 2). Closer examination with high-resolution TEM (HR-TEM) imaging reveals the “FeO*_x_*” nanoparticles/clusters as a minor component appearing mostly on the surface of MOF particles (Figures S5 and S6).

### 3.2 Continuous Methane Oxidation

**Figure 3:**
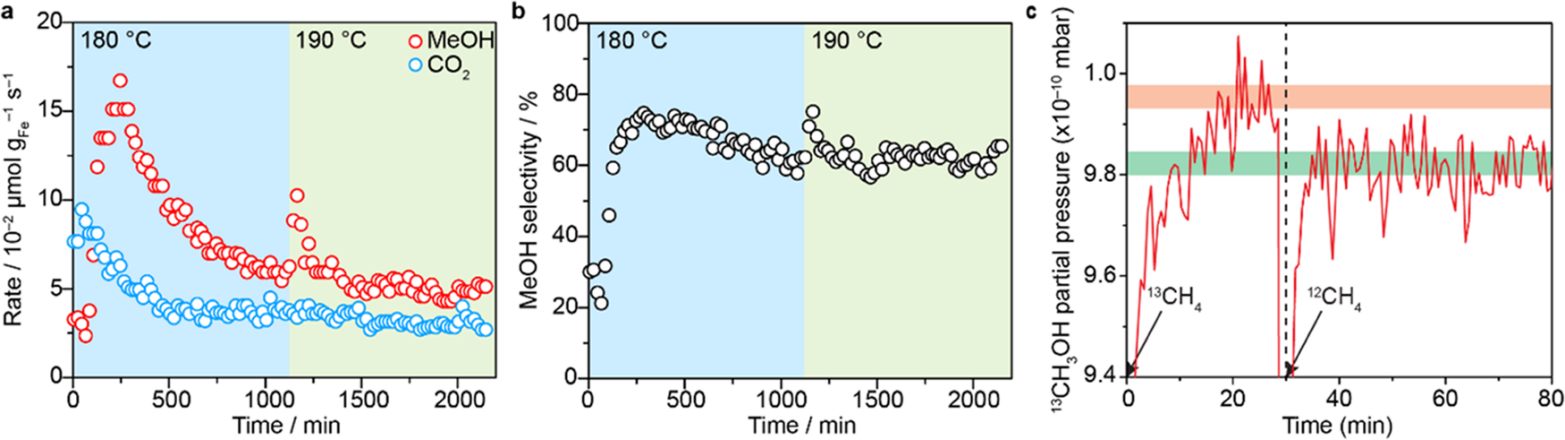
On-stream oxidation of methane to methanol over Fe/UiO-66. (a) Rates of methanol and CO_2_ formation during the direct activation of CH_4_ (10% CH_4_, 5% O_2_ + 0.2% H_2_O, balance Ar) at 5 bar and 180 and 190 °C. (b) Product selectivity toward methanol formation ([MeOH]/[MeOH] + [CO_2_]). (c) Transient isotopically labeled methane oxidation at 1 bar and 180 °C. Dashed line refers to the point of switch from ^13^CH_4_ to ^12^CH_4_ during reaction.

The Fe/UiO-66 catalyst was first activated by heating up from room temperature to 250 °C under Ar to remove volatile adsorbates and exposed to (i) 10% O_2_/Ar at 250 °C for 1 h, (ii) 5% water vapor in Ar for 1 h at 250 °C, 1 bar and (iii) Ar while decreasing the temperature to the reaction temperature of 180 °C. This sequence of pretreatment conditions was found to be crucial in activating the catalyst, presumably through the conversion of the Fe sites into the active form (see discussions in Section 3.4). However, the effects of the activation conditions on the kinetic properties lie outside the scope of this study. Kinetic measurements were performed directly after the in situ activation of the catalyst in a flow microreactor (Figure S9). The gas mixture was subsequently introduced to the reactor (10% CH_4_, 5% O_2_, 0.2% H_2_O, balance Ar) while the pressure was increased from 1 to 5 bar within 5 min. Oxygen and water vapor were fed into the reactor to function as an oxidant and a methanol extractor, respectively.^17^ During the reaction, only methanol and carbon dioxide were observed as the products with the CH_4_ conversion in the range of 0.13–0.06% (Figures 3, S10, and S11). In the first 250 min, the methanol formation rate increases continuously from 3.3 × 10^–2^ to 16.7 × 10^–2^ μmol_MeOH_ g_Fe_^–1^ s^–1^ (Figure 3a). This initial activation phase is likely due to the change in the reaction site governed by the mobility of the Fe sites and the rearrangement in the local environment of the isolated Fe sites such as the partial replacement of Cl^–^ by OH^–^ ligands.^17^ Similar induction period was observed for Cu-ZSM-5 where the induction period precedes the highest methanol productivity. This phenomenon was ascribed due to the mobility of Cu sites and reconfiguration of the Cu structures.^17^

Then, the catalyst proceeds into a deactivation phase over 850 min on stream and eventually reached a sustained activity of 5.9 × 10^–2^ μmol_MeOH_ g_Fe_^–1^ s^–1^. We attribute this partial loss of catalytic activity to the aggregation of Fe sites during the reaction as described in the XAS section below. On increasing the reaction temperature to 190 °C, we observed an initial increase of catalytic activity within the first 5 min to 10.3 × 10^–2^ μmol_MeOH_ g_Fe_^–1^ s^–1^ followed by decay over 150 min. Then, the catalyst showed a stable activity of 5.0 × 10^–2^ μmol_MeOH_ g_Fe_^–1^ s^–1^. This initial increase is attributable to the accelerated thermal desorption of the methanol already performed at 180 °C. The reaction rate at 190 °C is similar to that at 180 °C presumably because the expected enhanced reaction rate is counteracted by the reduction of the number of active sites through the aggregation of isolated Fe sites into FeO*_x_* particles at higher temperatures (see further discussion in Section 3.3).

Regarding the total oxidation of methane to CO_2_, the rate of CO_2_ formation reached 9.5 × 10^–2^ μmol_CO_2__ g_Fe_^–1^ s^–1^ within 45 min on stream, and then the rate dwindled to 3.5 × 10^–2^ μmol_CO_2__ g_Fe_^–1^ s^–1^ over 650 min (Figure 3a). Upon increasing the temperature to 190 °C, the CO_2_ production rate is not affected by the change in the reaction temperature. It should be noted that the reaction profiles in the first 500 min for the formation of methanol and CO_2_ follow a different pattern implying that the catalytic sites producing these products are different. Given that Fe_2_O_3_, which is also presented as an impurity in the catalyst, could oxidize methanol to carbon dioxide,^36^ it is probable that methanol produced by isolated Fe sites is subsequently converted to carbon dioxide by Fe_2_O_3_ nanoparticles. The decreased CO_2_ production rate is likely due to the aggregation of Fe_2_O_3_ nanoparticles during reaction on stream. Under the steady-state conditions at 180 °C, the selectivity toward methanol is 62% (Figure 3b). A similar catalytic activity measurement was carried out on a fresh catalyst at 150 °C following the activation protocol described above (see Figure S12). The results showed significantly lower activity for both methanol and CO_2_ formation. The activation phase was however rather shorter compared to the measurement done at 180 °C.

Additionally, we performed several controlled experiments to verify the source of the reaction products observed. First, a similar experiment performed on an inert material (SiO_2_) under identical reaction conditions shows no catalytic activity suggesting that the formation of methanol and CO_2_ is associated with Fe/UiO-66 catalyst. Second, transient isotopically labeled methane oxidation using ^13^CH_4_ as a reactant (Figure 3c) was carried out at 1 bar. This experiment exhibits the signal of ^13^CH_3_OH (*m*/*z* = 33) which decreases to the baseline level after switching the feed from ^13^CH_4_ to ^12^CH_4_ at 30 min of reaction on stream. This result suggests that methanol is generated by the oxidation of methane not from the decomposition of the catalyst. Furthermore, the ^12^CH_3_OH and ^12^CO_2_ signals during steady-state partial methane oxidation over Fe/UiO-66 are significantly higher than that over UiO-66 which appear at the baseline level substantiating the role of Fe sites in catalyzing the methane oxidation (Figure S14). Lastly, we carried out the methane oxidation over Fe/UiO-66 catalyst in the absence of one of the reaction components (i.e., CH_4_, O_2_, or H_2_O) and tracked the ^12^CH_3_OH signal (Figure S15). Under the feed of 10% CH_4_ + 5% O_2_ + 0.2% H_2_O, we observed a steady production of ^12^CH_3_OH. However, removal of either of those feeds results in a drop of ^12^CH_3_OH signal to the baseline level confirming the need for the coexistence of all of these gases. These results together indicate that methanol is produced from methane over Fe/UiO-66 catalyst.

In terms of the catalyst performance, Fe/UiO-66 exhibits a moderate methanol production rate of 2.0 × 10^–3^ μmol_MeOH_ g_cat_^–1^ s^–1^ compared to Cu-exchanged zeolites. However, it stands out as the first Fe-based catalyst that utilized O_2_ as an oxidant for partial oxidation of methane in gas phase (Table S3).^17,18,20,37−42^ Finally, to examine the possibility of reverting the observed deactivation, we attempted to reactivate the catalyst following the pretreatment protocol described above. The reactivated catalyst shows the methanol formation rate of 4.8 × 10^–2^ μmol_MeOH_ g_Fe_^–1^ s^–1^ after 5 min which gradually dropped to a steady-state value of about 2.5 × 10^–2^ μmol_MeOH_ g_Fe_^–1^ s^–1^ (Figure S13) at 180 °C and remained unchanged over 500 min. This result indicates that the deactivation of the initial activity detected after activation of the fresh catalyst is irreversible. After the reaction, we examined the integrity of the UiO-66 framework using PXRD which showed a similar diffraction pattern to the as-synthesized Fe/UiO-66 highlighting the structural stability of the MOF (Figure S16).

### 3.3 Structure Identification of the Fe Active Sites

To characterize the identity of “FeO*_x_*” nanoparticles and possible correlation with the observed catalyst deactivation, we performed high-resolution TEM imaging (HR-TEM) on Fe/UiO-66. However, the “FeO*_x_*” particles are too small to obtain sufficient crystallographic information. We, therefore, analyzed the nanoparticles on the pretreated catalyst where “FeO*_x_*” grew during the pretreatment step (Figures S17 and S19). Analysis of lattice fringes indicates that “FeO*_x_*” nanoparticles are of the Fe_2_O_3_ phase (Figure S18). Based on the presence of the Fe_2_O_3_ nanoparticles after the reaction, the catalyst deactivation was thus ascribed to the agglomeration of isolated Fe sites into Fe_2_O_3_ nanoparticles during the reaction on stream (Figures S20 and S21).

**Figure 4:**
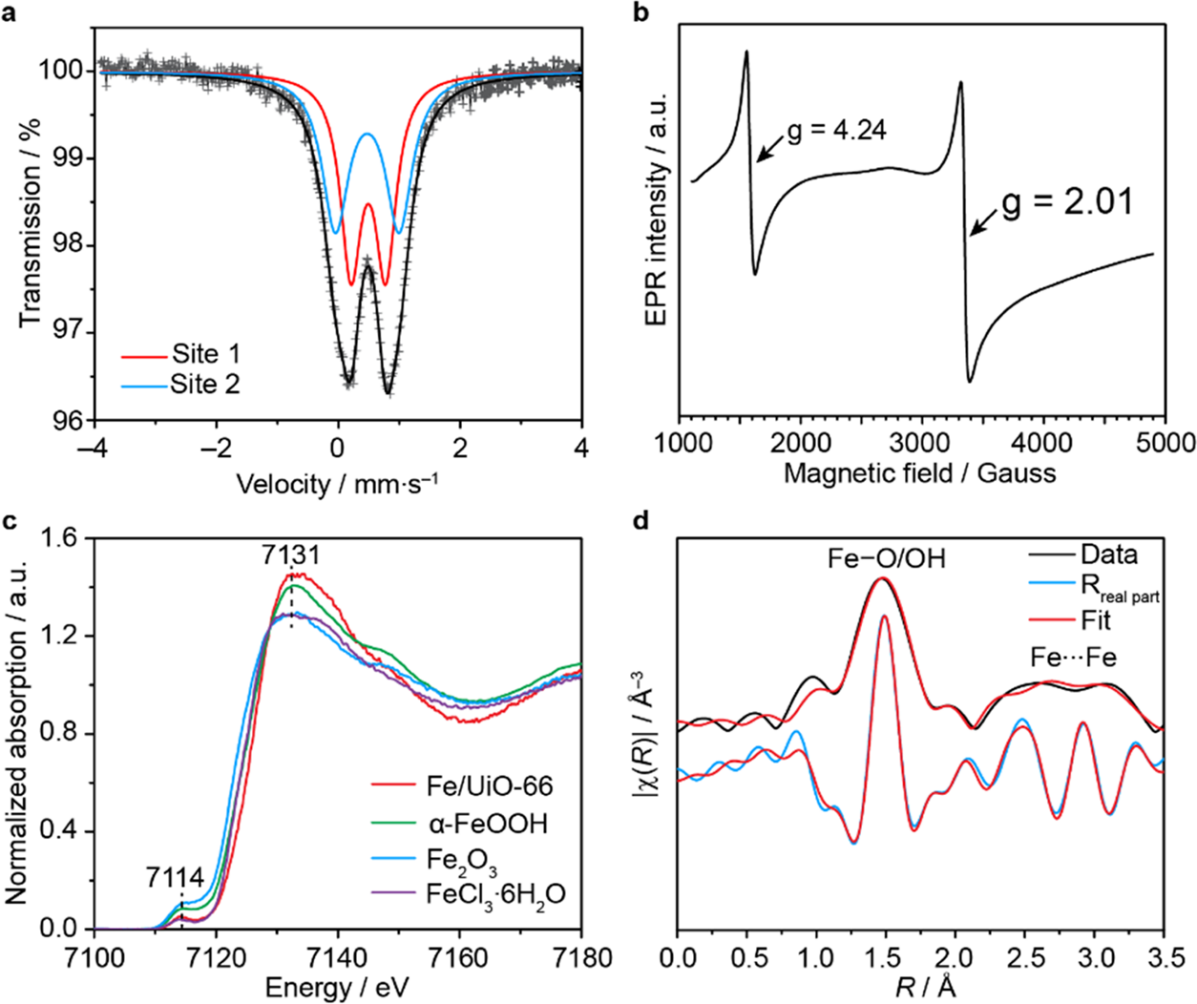
Structural analysis of the Fe sites in Fe/UiO-66. (a) Mössbauer spectrum of the as-synthesized Fe/UiO-66 collected at 80 K plotted as gray crosses. The fit to the spectrum is shown in black solid line. (b) EPR spectrum of the as-synthesized Fe/UiO-66 collected at 140 K. (c) Fe K-edge XANES spectra of as-synthesized Fe/UiO-66 overlaid with that of standard compounds measured under ambient conditions. (d) Fourier transformed Fe K-edge EXAFS spectrum of the as-synthesized Fe/UiO-66 catalyst.

To further elucidate the structures of the Fe species in the catalyst, we collected ^57^Fe Mössbauer spectra of the as-synthesized catalyst at 5 and 80 K. The 80 K spectrum can be fit to two symmetric Lorentzian doublets with isomer shifts (δ) of 0.474(1) and 0.492(1) mm s^–1^ and quadrupole splitting (Δ*E*_Q_) of 1.05(1) and 0.571(7) mm s^–1^, respectively (Figure 4a). These parameters are consistent with high-spin Fe(III) species in an octahedral environment coordinated by oxygen atoms.^43^ The relatively large quadrupole splitting of the first site also infers distortion of this octahedral Fe site. The relative ratio of the two Fe sites is ∼1:1. The 5 K spectrum can be fit to a combination of a symmetric quadrupole doublet and a magnetic hyperfine sextet (Figure S22). The quadrupole doublet features an isomer shift of 0.513(4) mm s^–1^, with a quadrupole splitting of 0.863(7) mm s^–1^, again consistent with a high-spin Fe(III) in an octahedral environment. Additionally, this result is in accordance with the computational results in which a high-spin state (*S* = 5/2; sextet state) is calculated to be the ground state of Fe/UiO-66 (Section S9). The observation of magnetic hyperfine sextet indicates magnetic ordering in one of the two Fe sites, characteristic of long-range magnetic ordering in iron oxide nanoparticles. This sextet features an isomer shift of 0.453(7) mm s^–1^, a quadrupole splitting |Δ*E*_Q_| = 0.05(1) mm s^–1^, and an internal magnetic field of 47.86(6) mT, the parameters of which are associated with Fe_2_O_3_.^44^ This result supports the presence of Fe_2_O_3_ nanoparticle as observed in the HR-TEM data described in the previous section. Fitting 5 K spectrum gives a relative area of 54.1(7)% Fe_2_O_3_ and 45.9(4)% high-spin Fe(III) center (Table S4).

To further investigate the electronic nature of these Fe species, an EPR spectrum of Fe/UiO-66 was measured under a continuous wave, X-band frequency at 9.40 GHz, 140 K. Two signals were identified with an effective *g* value of 2.01 and 4.24 (Figure 4b). The *g* = 2.01 signal is attributed to the ferrimagnetic resonance of γ-Fe_2_O_3_, in good agreement with TEM and Mössbauer data.^45,46^ The *g* = 4.24 signal, on the other hand, is typical of a high-spin Fe(III) possessing a rhombic zero-field splitting.^47^ This signal was attributed to an isolated Fe(III) species in the distorted octahedral coordination as identified by Mössbauer spectroscopy. After the reaction, the intensity of both *g* = 2.01 and *g* = 4.24 signals decreases which is due to the agglomeration of these Fe (Figure S23). Overall, the Fe/UiO-66 catalyst contains two Fe species: isolated Fe(III) and Fe_2_O_3_ species.

Afterward, we performed Fe K-edge XAS to probe the electronic and structural properties and coordination environment of the Fe sites in the catalyst. In the XANES region, the preedge feature was observed at 7114 eV, which is correlated with 1s → 3d quadrupole transition and dipole-allowed transition mediated by 3d–4p orbitals mixing (Figure 4c). The location of this preedge energy is in the same region as Fe_2_O_3_ suggesting that the oxidation state of the Fe sites is +3.^48^ The edge transition (1s → 4p transition) is located at 7127 eV which is higher than absorption edges found for typical Fe(III) species reported for different Fe(III) environments including FeOOH (7125 eV), Fe_2_O_3_ (7123 eV), FeCl_3_·6H_2_O (7124 eV), Fe supported on Zr-based MOF synthesized by another method (7123 eV),^49^ and isolated Fe in MIL-53 (7126 eV).^27^ The data suggest that Fe in Fe/UiO-66 is electron-deficient which could correlate with the origin of the catalytic activity for the oxidation of methane. The pronounced preedge peak and nonintense white line indicate that the Fe(III) sites occupy tetrahedral sites or distorted octahedral geometry^50^ where the latter case is more likely as it is in agreement with the Mössbauer data. Based on XANES analysis, the oxidation state of the Fe sites after the pretreatment and methane oxidation steps remains +3 (Figure S24).

Analysis of the extended X-ray absorption fine structure (EXAFS) spectra of Fe/UiO-66 provides information regarding the coordination environment of the Fe sites in the as-synthesized, pretreated, and spent samples. The best fit is displayed in Figure 4d and the fit parameters are listed in Table 1. From the analysis of the as-synthesized sample, the nearest scattering shell is found at 1.94 ± 0.02 Å with the coordination number (CN) of 2.3 ± 0.3 and at 2.11 ± 0.03 Å with the CN of 2.3 ± 0.3 (Figure S25). This suggests that Fe is 6-coordinated bound to OH and H_2_O ligands, which agrees with the Mössbauer analysis. Additionally, the EXAFS data fit well to backscattering shells of Fe···Fe at 2.99 ± 0.03 and 3.16 ± 0.06 Å with the CN of 1.5 ± 0.2 and 0.8 ± 0.1, respectively. The slightly longer distance and the significantly lower CN of this scattering shell compared to those of γ-Fe_2_O_3_ (2.95 Å and CN = 4) suggest that the Fe sites in this catalyst are a conglomerate of isolated Fe species and Fe_2_O_3_ species (Figures S30 and S31). After the pretreatment and the reaction, the first shell coordination parameters of Fe remain similar to what is observed in the as-synthesized sample. In contrast, the CN of Fe···Fe backscattering shell increases (Figures S26–S29) implying the formation of the long-range order through the agglomeration of Fe sites to FeO*_x_* nanoparticles, in agreement with the TEM analysis.

**Table 1:** Fe K-Edge EXAFS Fitting Result of Fe/UiO-66

Ab–Sc pair^a^^a^	CN^b^^b^	*R*^c^^c^	DWF^d^^d^	*R*-factor^e^^e^
as-synthesized Fe/UiO-66				
Fe–O1	2.3 ± 0.3	1.94 ± 0.02	0.0008 ± 0.0028	0.01
Fe–O2	2.3 ± 0.3	2.11 ± 0.03	0.0041 ± 0.0053	
Fe···Fe1	1.5 ± 0.2	2.99 ± 0.03	0.0034 ± 0.0041	
Fe···Fe2	0.8 ± 0.1	3.16 ± 0.06	0.0034 ± 0.0064	
pretreated Fe/UiO-66				
Fe–O1	2.5 ± 1.6	1.98 ± 0.08	0.0027 ± 0.0071	0.02
Fe–O2	2.5 ± 1.6	2.04 ± 0.17	0.0140 ± 0.0418	
Fe···Fe1	0.9 ± 0.5	2.93 ± 0.17	0.0153 ± 0.0248	
Fe···Fe2	1.7 ± 1.1	3.21 ± 0.25	0.0098 ± 0.0443	
spent Fe/UiO-66				
Fe–O1	2.1 ± 0.1	1.93 ± 0.01	0.0005 ± 0.0014	0.003
Fe–O2	2.1 ± 0.1	2.08 ± 0.01	0.0031 ± 0.0025	
Fe···Fe1	1.4 ± 0.1	2.97 ± 0.02	0.0046 ± 0.0022	
Fe···Fe2	2.1 ± 0.1	3.13 ± 0.03	0.0085 ± 0.0024	

a Ab = absorber; Sc = scatterer.

b Coordination number.

c Distance (Å).

d Debye–Waller factor (Å^2^).

e A measure of mean square sum of the misfit at each data point. Fit range: 2.5 < *k* < 11 Å^–1^; 1 < *R* < 3.8 Å. Fit window: Hanning.

XPS analysis was used to further inspect the electronic properties of the catalyst surface by measuring the Fe 2p, Cl 2p, and Zr 3d regions on the pretreated and the spent samples after the reaction over 1000 min at 5 bar and 180 °C (Figure S32). In the Fe 2p region, the main peak can be deconvoluted to two main components with binding energies of 711.0 and 713.9 eV. While the first component can be assigned to Fe^3+^ residing in Fe_2_O_3_ or FeOOH species, the second component is likely associated with the Fe^3+^ bound to strong electron-withdrawing ligands, in agreement with the XANES analysis. A strong peak at 719.3 eV was seen both on the pretreated spent catalysts, which can be assigned to the Fe 2p satellite confirming the presence of Fe^3+^ species.^51^ In the spent sample, we observed a similar spectral profile except for the binding energy of the first component of the Fe^3+^ peak which is slightly shifted to 710.7 eV. For Cl 2p, the peaks were observed at the same binding energies both in the pretreated sample and the spent sample. For Zr 3d, the binding energy changed from 182.9 eV in the as-synthesized sample to 182.7 eV in the spent sample which hints at a slight reduction of Zr interface sites after the reaction. Using the obtained peak areas of Fe, Zr, and Cl peaks and the corresponding atomic sensitivity factors, the surface atomic ratio of Fe/Zr was found to decrease from 0.34 in the pretreated sample to 0.14 in the spent sample (Figure S33), which agrees well with the interpretation of isolated Fe^3+^ sites partially aggregating to Fe_2_O_3_ during the reaction as previously deduced from TEM and EXAFS analysis.

### 3.4 Nature of the Active Sites and Reaction Intermediates

**Figure 5:**
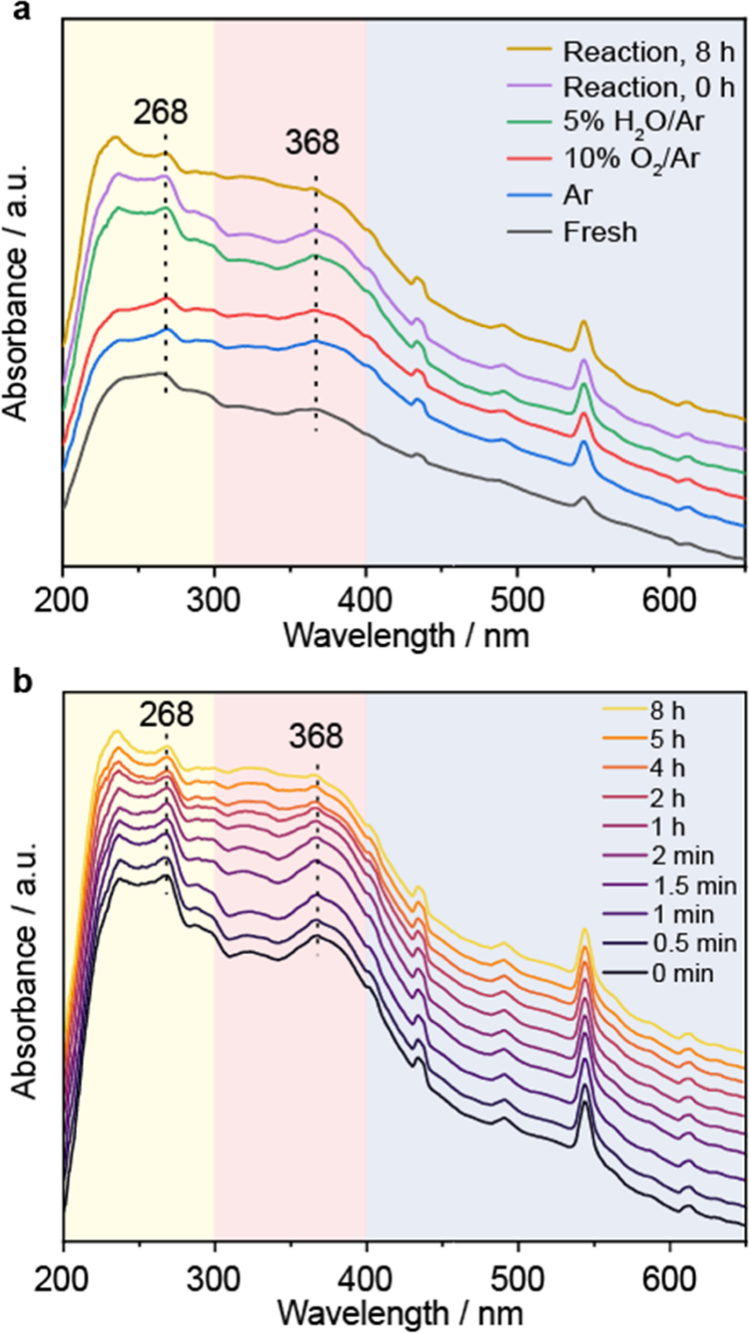
In situ UV–vis spectra of 3.1Fe/UiO-66 catalyst. (a) After each gas treatment including Ar at 250 °C, 1 h; 10% O_2_/Ar at 250 °C, 1 h; 5% H_2_O/Ar at 250 °C, 1 h; and 10% CH_4_ + 5% O_2_ + 2% H_2_O/Ar at 180 °C and (b) during the methane oxidation reaction.

To understand the evolution of the Fe sites during the reaction, we carried out in situ DR UV–vis spectroscopy because it is sensitive to the geometric nature of Fe species.^52,53^ UV–vis spectra of Fe(III) samples can be divided into three main regions: 200–300 nm for mononuclear Fe, 300–400 nm for binuclear bridged Fe-oxo species, and >400 nm for large Fe_2_O_3_ particles.^54−56^ In the fresh Fe/UiO-66 sample, peaks located at 268 and 368 nm as well as broadband absorption at >400 nm were observed (Figure 5a). Thus, the catalyst contains a mixture of mono-, bi-, and polynuclear Fe species. The intensity of the 368 nm grew slightly after Ar and 10% O_2_/Ar treatments and is more pronounced after 5% H_2_O/Ar treatment. During the methane oxidation reaction at 180 °C (Figure 5b), we observed a gradual decay of both 268 and 368 nm bands, especially after 1 h on stream. Based on the kinetic measurements where the catalytic activity decreased at extended reaction time, such mono- and binuclear could be the reaction sites for methane oxidation. The loss of mono- and binuclear species likely occurs through their agglomeration into larger FeO*_x_* nanoparticles.

To gain insight into the reaction mechanism of this catalyst, we performed in situ infrared spectroscopy measurements (see Figure S34 and details therein) to clarify probable active species on the catalyst surface during the reaction. The measurement was conducted employing the same procedure described for the kinetic measurements (10% CH_4_, 5% O_2_ + 0.2% H_2_O, balance Ar at 5 bar and 180 °C). In the C–H stretch region, we could resolve a distinct peak at 2858 cm^–1^, which can be assigned to the asymmetric C–H stretch of surface methoxy species (see Figure S35).^57−60^ Additionally, weaker peaks at 2829 and 2811 cm^–1^ are related to the C–H stretch and the combination of asymmetric and symmetric vibrational modes of COO, respectively, of formate-related species such as formate, formaldehyde, and formic acid.^60−62^ These species are likely relevant surface intermediates that will form a basis for future mechanistic study of this reaction.

## 4 Conclusions

We reported the synthesis and application of Fe supported by UiO-66 as a highly active and selective catalyst for on-stream partial methane oxidation to methanol in the gas phase. Detailed spectroscopic characterizations indicate that isolated Fe sites catalyze the formation of methanol while Fe_2_O_3_ impurity presumably converts methanol to CO_2_. Based on in situ DRIFTS experiment, the methoxy species were found as likely intermediates toward methanol product.

## Supporting Information Available

Detailed catalyst synthesis and characterization results including NMR, surface area analysis, TGA, SEM, TEM, SXRD, Mössbauer spectroscopy, EPR, XAS, and XPS; kinetic measurements and additional results; and details on in situ UV–vis and DRIFTS (PDF)
