# Supplementary material for: Structural Evolution of Iron-Loaded Metal–Organic Framework Catalysts for Continuous Gas-Phase Oxidation of Methane to Methanol
Source: ACS Appl Mater Interfaces. 2023 May 23;15(22):26700–9. doi: 10.1021/acsami.3c03310 (PMC10251415; doi:10.1021/acsami.3c03310)
Supplement: Supplementary file 1 — am3c03310_si_001.pdf [file am3c03310_si_001.pdf]

Supporting Information

Structural Evolution of Iron-Loaded Metal–Organic Framework Catalysts for Continuous Gas-Phase Oxidation of Methane to Methanol

Bunyarat Rungtaweevoranit,[1]* Ali M. Abdel-Mageed,[2,3,4]* Pongtanawat Khemthong,[1] Srisin Eaimsumang,[1] Khetpakorn Chakarawet,[5,6] Teera Butburee,[1] Benny Kunkel,[2] Sebastian Wohlrab,[2] Kittipong Chainok,[7] Jakkapop Phanthasri,[1] Suttipong Wannapaiboon,[8] Saran Youngjan,[1] Theerada Seehamongkol,[1] Sarawoot Impeng,[1] and Kajornsak Faungnawakij[1]*

[1]National Nanotechnology Center (NANOTEC), National Science and Technology Development Agency (NSTDA), Pathum Thani 12120, Thailand

[2] Leibniz-Institut für Katalyse e.V. (LIKAT Rostock), Albert-Einstein-Str. 29a, 18059 Rostock, Germany

[3]Department of Chemistry, Faculty of Science, Cairo University, 12613 Giza, Egypt

[4]Institute of Surface Chemistry and Catalysis, Ulm University, D-89069 Ulm, Germany

[5]Department of Chemistry, University of California, Berkeley, CA 94720, USA

[6]Department of Chemistry, Faculty of Science, Mahidol University, Bangkok 10400 Thailand

[7]Thammasat University Research Unit in Multifunctional Crystalline Materials and Applications (TU-MCMA), Faculty of Science and Technology, Thammasat University, Pathum Thani 12121, Thailand

[8]Synchrotron Light Research Institute (Public Organization), 111 University Avenue, Suranaree, Muang, Nakhon Ratchasima, 30000 Thailand

*Corresponding authors

E-mail: bunyarat.run@nanotec.or.th, ali.abdelmageed@catalysis.de and kajornsak@nanotec.or.th

Section S1. Synthesis of Materials

Chemicals. Zirconium tetrachloride, 1,4-benzenedicarboxylic aicd, and *N*,*N*-diethylformamide (DEF) were obtained from Acros. Analytical grade *N*,*N*-dimethylformamide and acetone were purchased from RCI Labscan. Acetic acid (99.7%) was obtained from Loba Chemie. $FeCl_3 \cdot 6H_2O$ (97%) was purchased from Sigma-Aldrich. Formic acid was obtained from EMD Millipore. These chemicals were used without further purification.

Catalyst synthesis

UiO-66. UiO-66 was synthesized following the reported protocol.[1] Terephthalic acid and zirconium tetrachloride were dissolved in a 20 mL vial containing DMF (10 mL) and acetic acid (0.7 mL). The vial was sealed and heated in a convection oven preheated at 120 °C for a day. White powder was collected by centrifugation (10,000 rpm, 5 min), washed 3 times with DMF (5 mL × 3) over 24 h period, and 3 times with acetone (5 mL × 3) over a 24 h period. Finally, UiO-66 was dried under dynamic vacuum overnight at room temperature.

Fe/UiO-66. UiO-66 (600 mg) was added to a solution containing $FeCl_3 \cdot 6H_2O$ (856 mg) dissolved in DMF (9 mL). The suspension was sonicated for one minute. The vial's thread was wrapped with PTFE tape, sealed and heated in an 85 °C isothermal oven for 15 h. The yellow-orange product was collected by centrifugation (10,000 rpm, 5 min), washed with DMF for 5 times (25 mL × 5) over 3 d period, and acetone for 3 times (25 mL × 3) over 24 h period. The sample was dried under dynamic vacuum overnight at room temperature.

The amount of Fe in the catalyst was analyzed by an inductively coupled plasma atomic emission spectroscope (ICPE-9820, Shimadzu). The sample (~4 mg) was digested in a solution mixture of H_2SO_4 (1.5 mL, 98%) and H_2O_2 (0.5 mL, 30 wt.% in H_2O) by heating in a microwave reactor (MiniWAVE, SCP SCIENCE) at 180 °C for 10 min at a ramp rate of 15 $°C \cdot min^{-1}$. The resulting solutions were diluted with milliQ water to 25 mL before the measurement.

Synthesis of single crystalline sample

Single crystals of UiO-66. Following the reported procedure,[2] zirconium oxychloride octahydrate (12 mg) was dissolved in DEF (1 mL). Separately, terephthalic acid (5 mg) was dissolved in DEF (1 mL). The solutions were mixed together in a 20-mL scintillation vial and formic acid (2 mL) was added. The resulting solution was shaken and placed in a 120 °C isothermal oven for 2 days. The crystals were washed with DMF 5 times (5 mL × 5) over 3 days.

Single crystals of Fe/UiO-66. Approximately 20 mg of single crystals of UiO-66 was added to a solution of $FeCl_3 \cdot 6H_2O$ (856 mg) dissolved in DMF (9 mL) in a 20-mL scintillation vial. The reaction was sealed and heated in an 85 °C isothermal oven for 15 h. The crystals were washed

with DMF 5 times (10 mL×5) over 3 days and with acetone 5 times (10 mL×5) over 3 days. Finally, the crystals were dried under dynamic vacuum at 120 °C overnight.

Section S2. Materials Characterization

Section S2.1 Solution ^{1}H NMR of digested samples

After the solvent exchange step, the samples were dried under dynamic vacuum at 150 °C overnight prior to the digestion which was achieved by sonicating the samples (5 mg) in a mixture of DMSO-d_6 (560 µL), hydrofluoric acid 48% (20 µL) and D_2O (20 µL). ^{1}H NMR spectra of the digested samples were recorded on a Bruker Advance III 400 (400 MHz) spectrometer or Bruker Ultrashield Plus 500 (500 MHz) at 297–300 K.

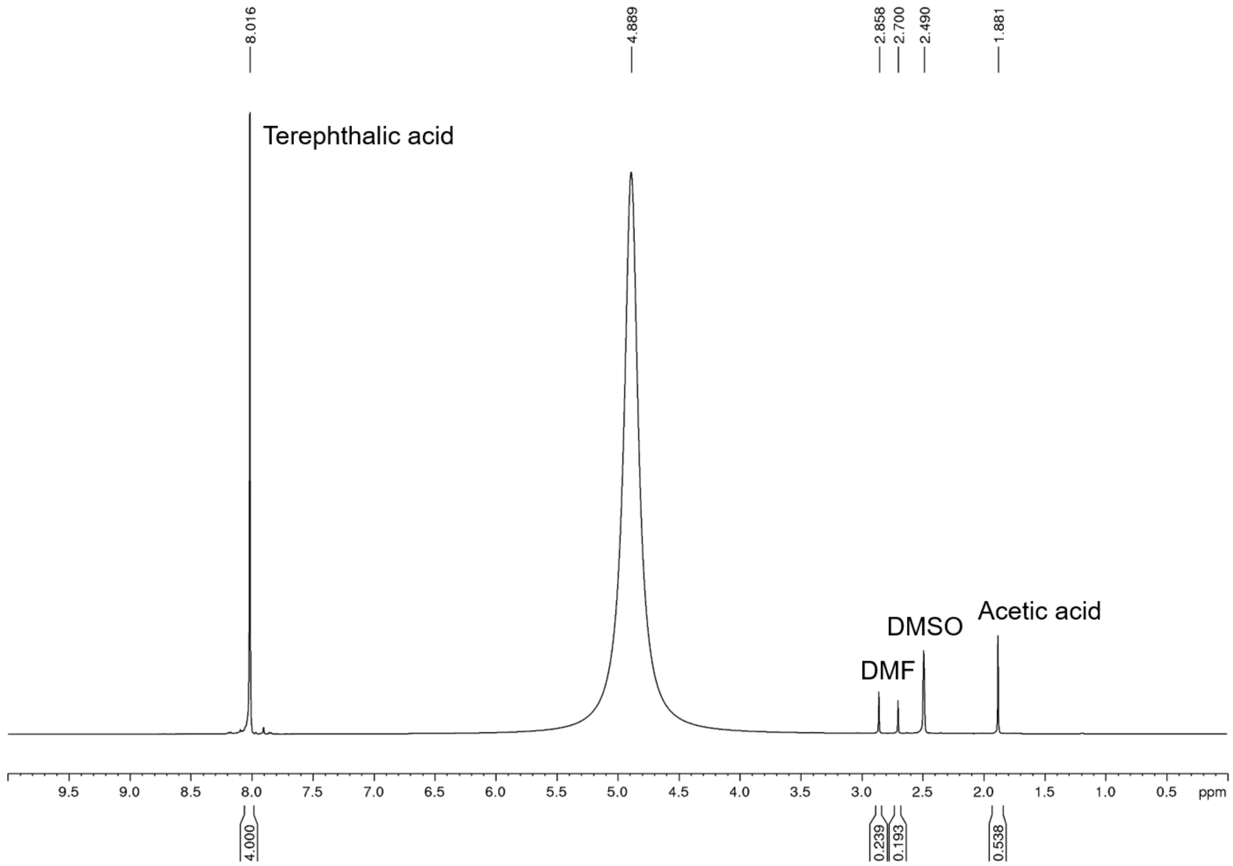

Figure S1. ^{1}H NMR spectrum of digested UiO-66 showing the presence of acetic acid and terephthalic acid in a molar ratio of 0.18:1.

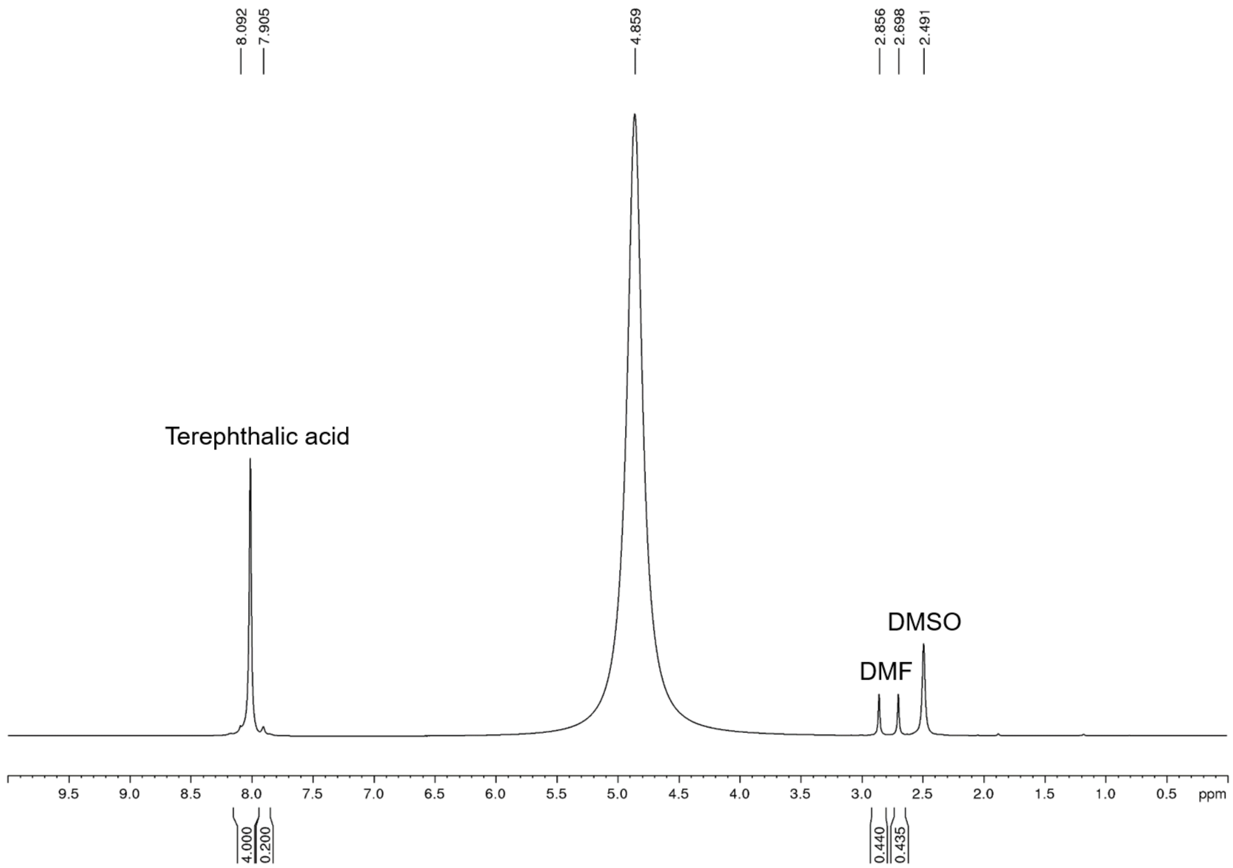

Figure S2. ^{1}H NMR spectrum of digested Fe/UiO-66 showing the removal of acetic acid from the structure during the installation of Fe sites.

Section S2.2 Powder X-ray diffraction

Powder X-ray diffraction patterns (PXRD) were recorded using a Bruker D8 Advance diffractometer (Bragg-Brentano, monochromated Cu Kα radiation $\lambda = 1.54056$ Å).

Section S2.3 Nitrogen sorption measurements

Gas adsorption analyses were conducted on a Quantachrome iQ-MP/XR volumetric gas adsorption analyzer. A liquid nitrogen bath (77 K), ultra-high purity grade N_2 and He (99.999%, Praxair) were used for the measurements. The samples were heated in a pre-weighted sample tube to 150 °C under dynamic vacuum with a ramp rate of 5 °C/min and kept at this temperature until an outgas rate of less than 50 mTorr/min was reached. The samples were cooled down naturally to room temperature under dynamic vacuum and filled with N_2 gas. The sample tubes were measured using an electronic balance to determine the sample weights and transferred to analysis ports.

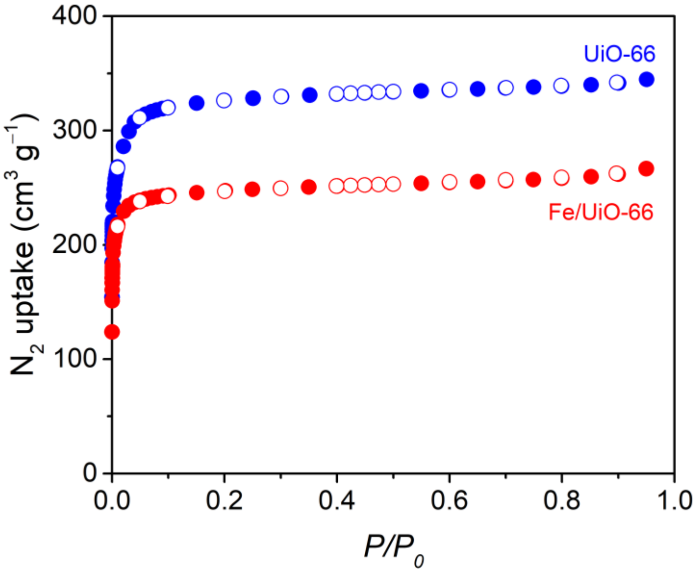

Figure S3. Nitrogen sorption isotherms of UiO-66 and Fe/UiO-66

Table S1. Summary of surface areas of the materials presented in the graphs shown above.

Material	Calculated BET surface area ($m^2\ g^{-1}$)
UiO-66	1326
Fe/UiO-66	1010

Section S2.4 Thermal gravimetric analysis

Thermal gravimetric analysis (TGA) was performed using a Mettler Toledo TGA/DSC 3+ HT/1600 thermal gravimetric analyzer under air flow and a heating rate of 5 °C min^{-1}. The samples were used directly after the solvent exchange in acetone and dried under dynamic vacuum at room temperature. The mass loss below 120 °C is thus attributed to the loss of physisorbed water molecules.

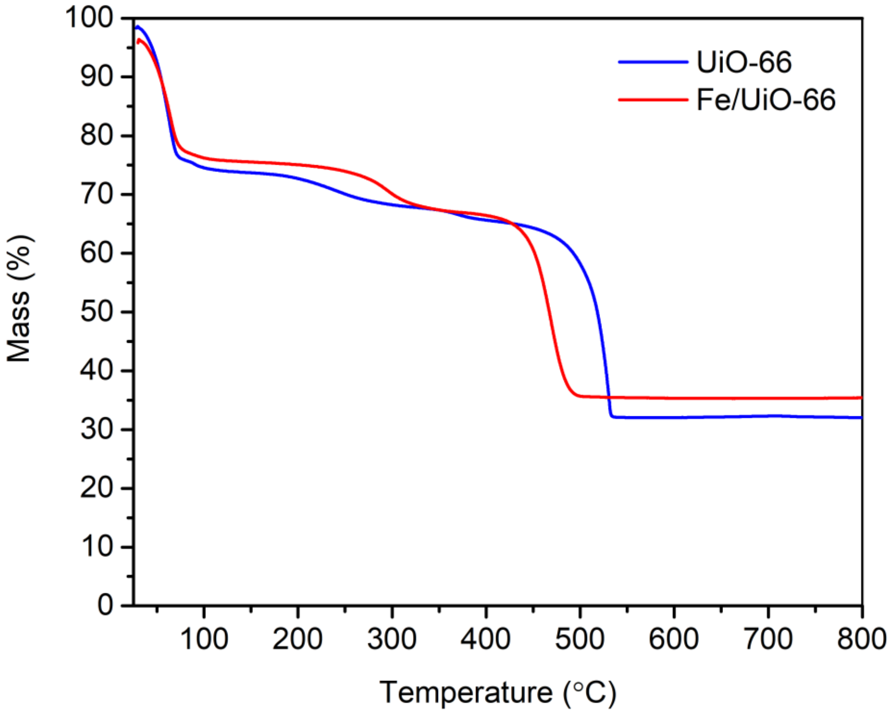

Figure S4. TGA trace of UiO-66 and Fe/UiO-66 showing that Fe/UiO-66 is stable in air up to 300 ºC.

Section S2.5 Scanning electron microscopy (SEM)

Scanning electron microscope (SEM) images were obtained using a Hitachi SU8030 scanning electron microscope. The sample was prepared by dispersing MOF samples in ethanol by sonication and the samples were drop-casted on a silicon wafer.

Section S2.6. Transmission electron microscopy

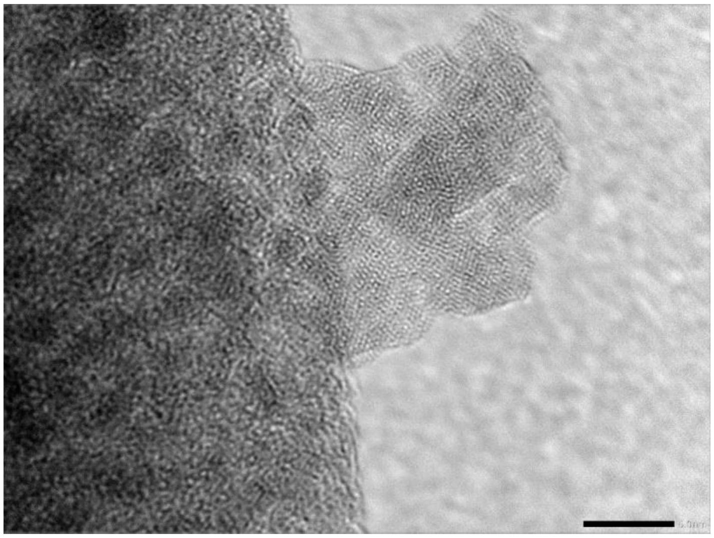

Figure S5. HR-TEM image of as-synthesized Fe/UiO-66 focusing at ‘FeO_x’ nanoparticle.

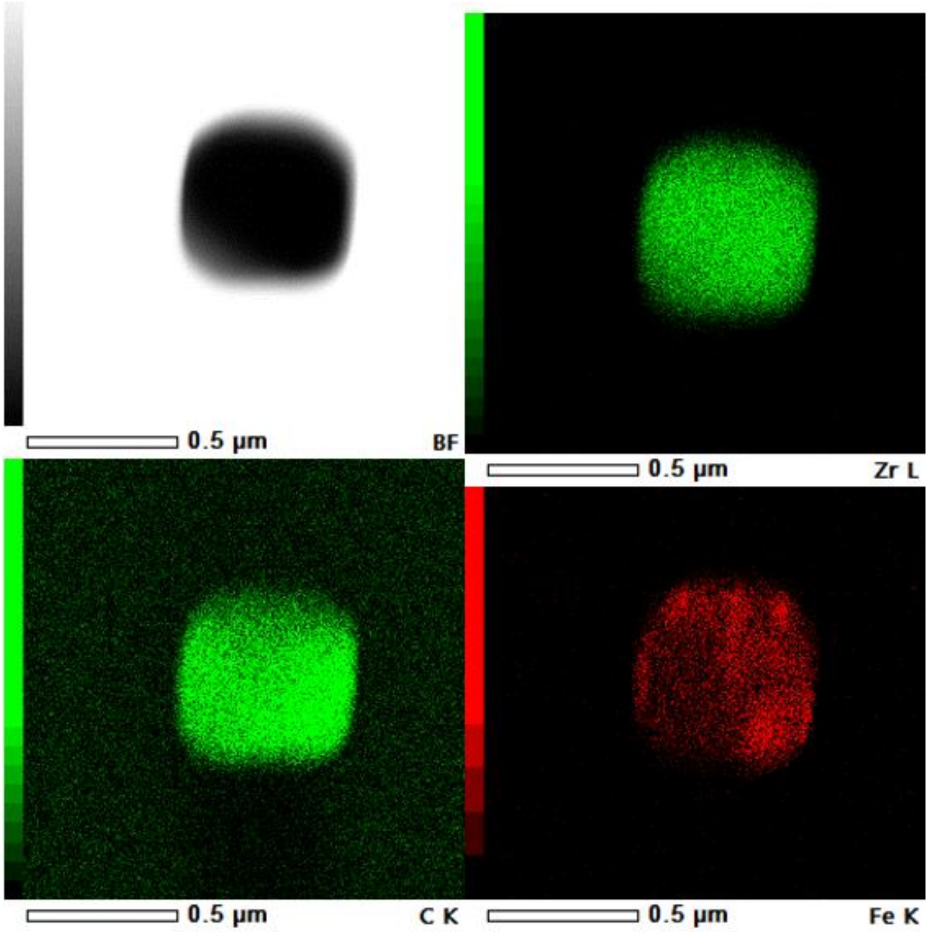

Figure S6. Elemental mapping of as-synthesized Fe/UiO-66 using TEM-EDS.

Section S3. Single-crystal x-ray diffraction analysis

Single-crystal X-ray diffraction studies revealed that the compound crystallizes in the cubic space group *Fm*-3*m* and displays a three-dimensional framework.[3] Apparently, the structure was found to be identical to that reported by the previous single-crystal X-ray determination in terms of the establishment of the host framework topology and the defect sites between the organic linkers and/or water and hydroxy groups (Figure 1b). Despite our attempts to locate the iron atoms, all our efforts proved fruitless due to the highly disordered and/or the defection of organic linkers and the guest solvent molecules as well as low concentrations of electron density of the Fe sites (see Figure S10).

X-ray diffraction data were collected using a Bruker D8 VENTURE CMOS PHOTON II with graphite monochromated Cu-Kα (λ = 1.54184 Å) radiation at 150(2) K. Data reduction was performed using SAINT (V8.38A).[4] Absorption correction were performed by using SADABS-2016/217.[4] The structure was solved with the ShelXT program,[5] and was refined by least squares using ShelXL[6] within the OLEX2 GUI.[7] The crystallographic details for all the complexes are summarized in Table S2.

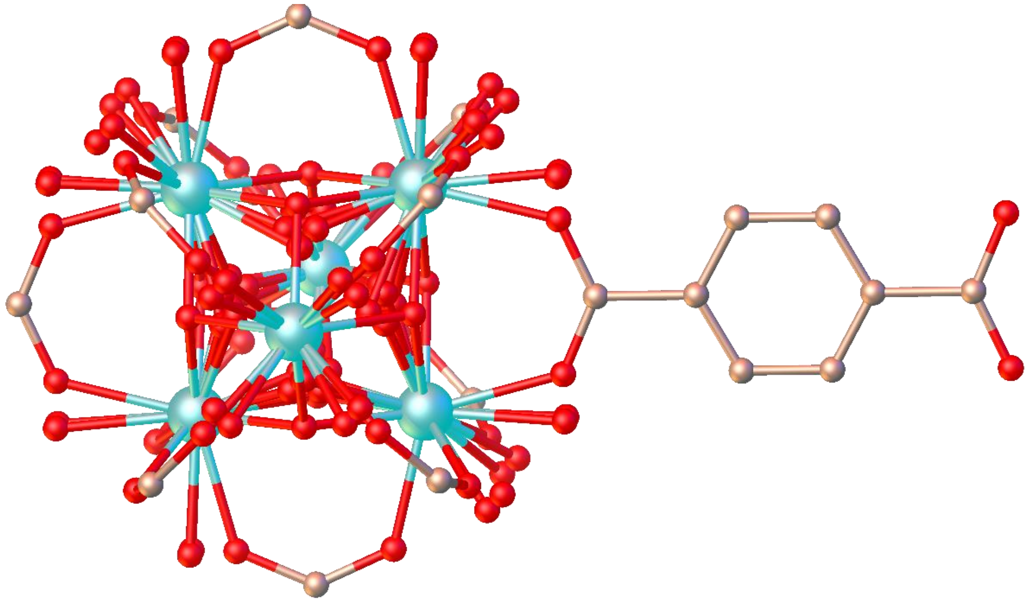

Figure S7. View of the defect sites between the organic linkers and/or water and hydroxy groups in Fe/UiO-66.

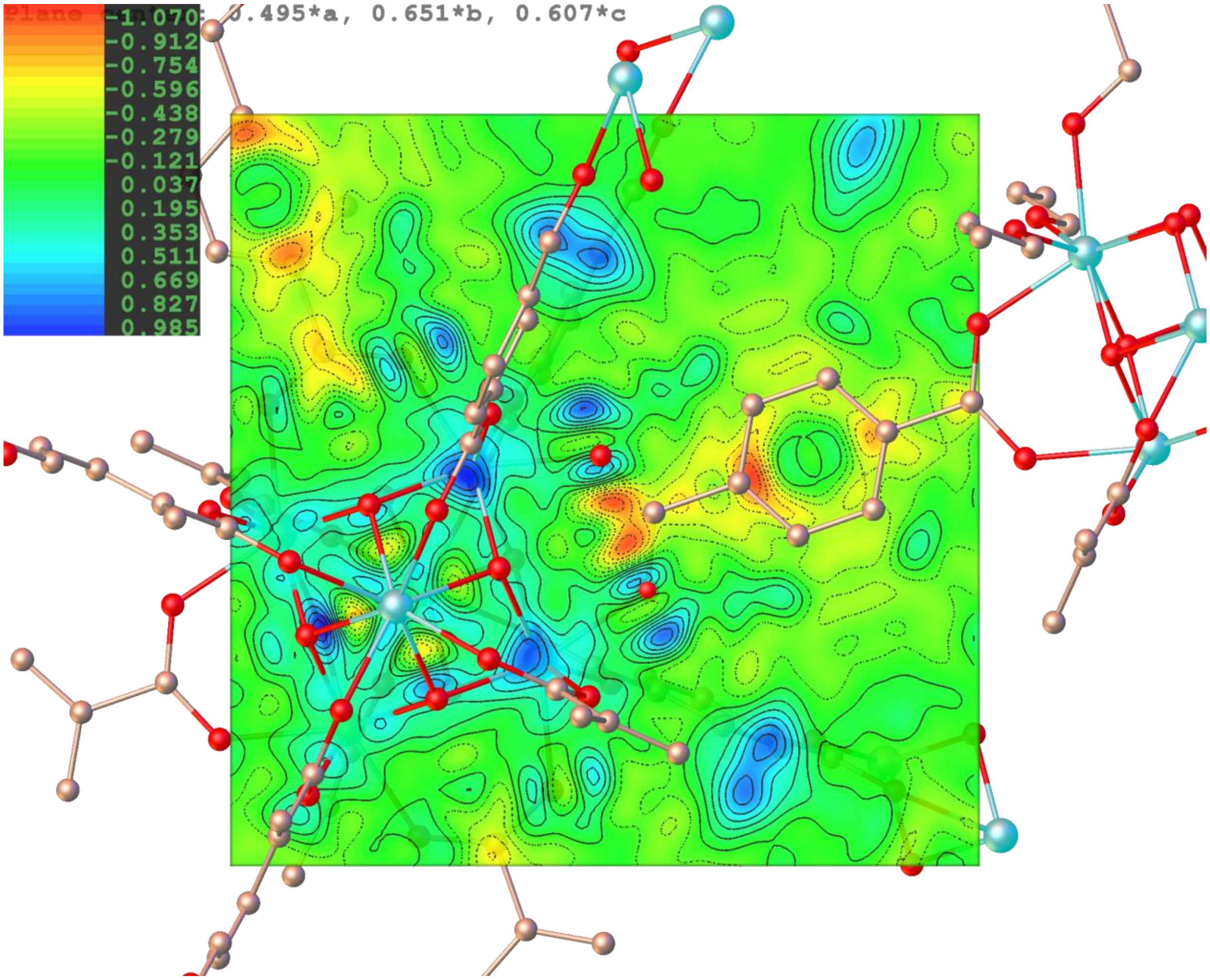

Figure S8. The differential electron density map of Fe/UiO-66 plotted on the plane of (0.495, 0.651, 0.607). The UiO-66 framework is fully refined; thus, the remaining electron densities are caused by the unassigned Fe site. Contour levels are labeled at the top left corner with the units in e/Å^3.

Table S2. Crystal data and structure refinement for Fe/UiO-66.

Empirical formula	$C_{12}H_8O_8Zr_{1.5}$
Formula weight	408.95
Temperature/K	150
Crystal system	cubic
Space group	*Fm*-3*m*
a/Å	20.7327(3)
b/Å	20.7327(3)
c/Å	20.7327(3)
α/°	90
β/°	90
γ/°	90
Volume/Å^3	8911.8(4)
Z	16
ρ_{calc}g/cm^3	1.219
μ/mm^{-1}	6.097
F(000)	3136.0
Crystal size/mm^3	0.22 × 0.20 × 0.20
Radiation	CuKα (λ = 1.54178)
2Θ range for data collection/°	7.384 to 144.476
Index ranges	$-25 \leq h \leq 10$, $-20 \leq k \leq 15$, $-25 \leq l \leq 18$
Reflections collected	5211
Independent reflections	500 [R_{int} = 0.0278, R_{sigma} = 0.0137]
Data/restraints/parameters	500/0/28
Goodness-of-fit on F^2	1.130
Final R indexes [I≥2σ (I)]	R_1 = 0.0441, wR_2 = 0.1469
Final R indexes [all data]	R_1 = 0.0450, wR_2 = 0.1484
Largest diff. peak/hole / e Å^{-3}	1.09/-1.09

Section S4. Kinetic measurements

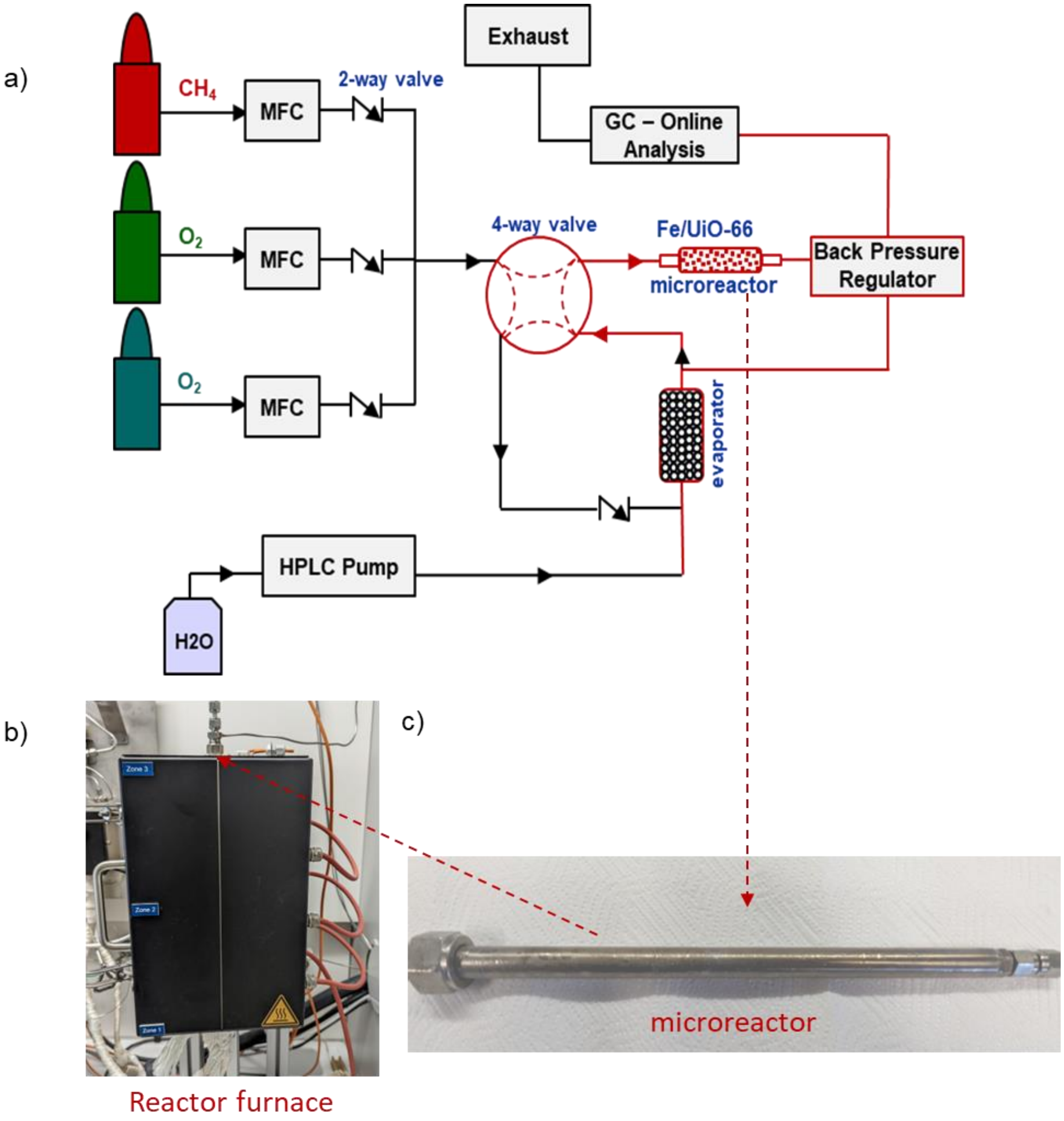

Figure S9. (a) Schematic representation of the reaction setup used in kinetic and in situ FTIR measurements (red lines reflects heating at 80 °C; MFC: Mass flow controller; HPLC: high performance liquid chromatography pump).(b) Image of the reaction furnace, and (c) stainless steel microreactor.

Table S3. Comparison of catalysts, reaction conditions and their catalytic activity for continuous gas-phase oxidation of methane to methanol.

Catalyst	Temperature (ºC)	Pressure (bar)	Reactant	Specific activity (μmol$_{MeOH}$ h^{-1} g$_{cat}$$^{-1}$)	MeOH selectivity (%)	Reference
Cu-Na-ZSM-5	210	1	CH_4, O_2, H_2O	0.88	71	[8]
Cu-CHA	270	1	CH_4, O_2, H_2O	5.35	55	[9]
Cu-CHA	300	1	CH_4, O_2, H_2O	195	85	[10]
Cu-CHA	300	1	CH_4, O_2, H_2O	366	22	[11]
Cu-SSZ-39	325	1	CH_4, N_2O, H_2O	499	34	[12]
Cu-SSZ-39	225	1	CH_4, O_2, H_2O	9.82	-	[13]
NiCuO/CZ	250	1	CH_4, O_2, H_2O	2.84	1.43	[14]
FeZSM-5	300	1	CH_4, N_2O, H_2O	52.5	62	[15]
Fe-BEA-1	250	1	CH_4, N_2O, H_2O	227	73	[16]
Fe/UiO-66	180	5	CH_4, O_2, H_2O	7.2	62	This work

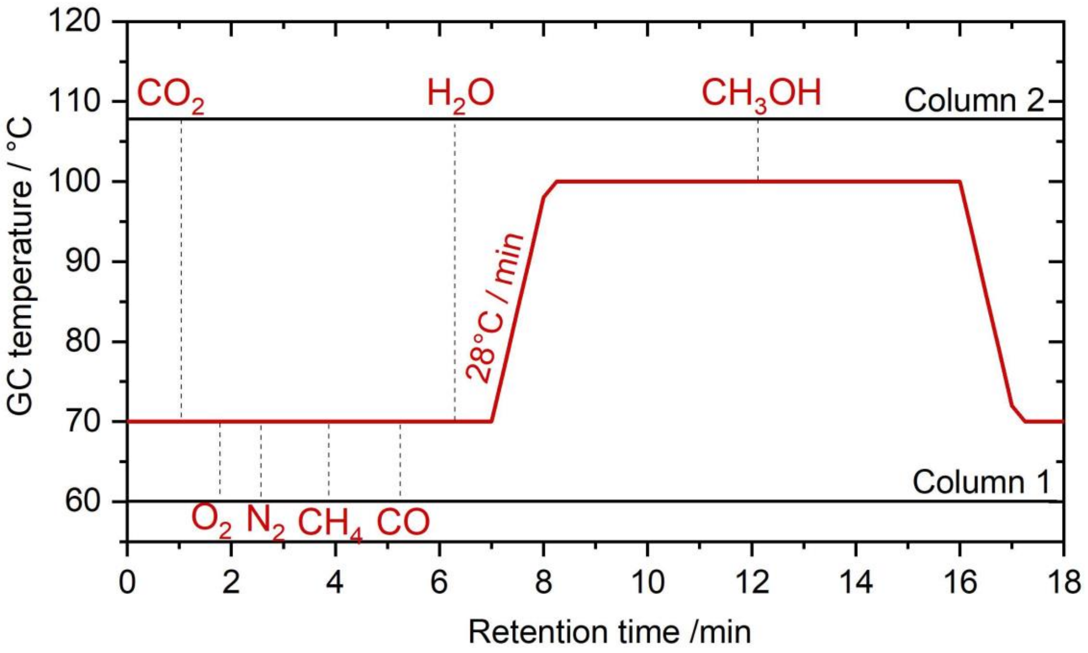

Figure S10. Schematic diagram showing the temperature program used in gas separation and the retention time for different gas components on column 1(Hayesep Q polymer) and on column 2 (molecular sieve 5 Å).

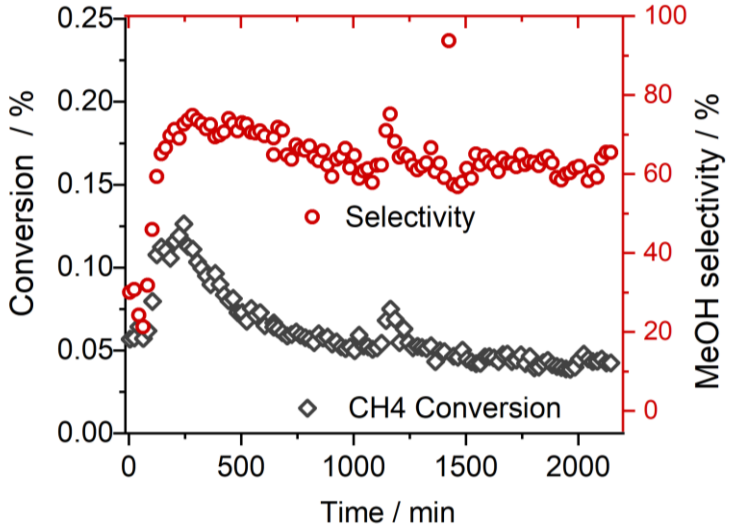

Figure S11. On-stream methane conversion during the direct activation of CH_4 (10% CH_4, 5% O_2 + 0.5% H_2O, balance Ar) at 5 bar and 180–190 °C.

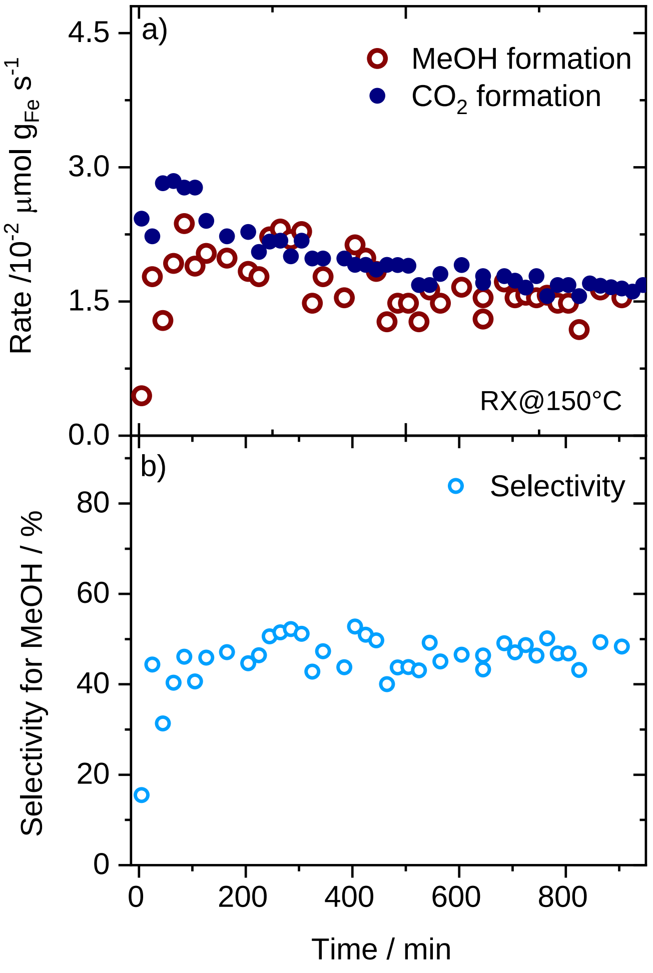

Figure S12 On-stream partial oxidation of methane to methanol 150 °C / 5bar (10% CH_4, 5% O_2 + 0.5 % H_2O)after activation step described in the experimental part . **a** Rate of formation of methanol (○) and CO_2 (●) during the direct activation of CH_4 at 5 bar and 180 °C. **b** Selectivity (○) toward methanol formation ([MeOH]/{[MeOH] + [CO_2]}).

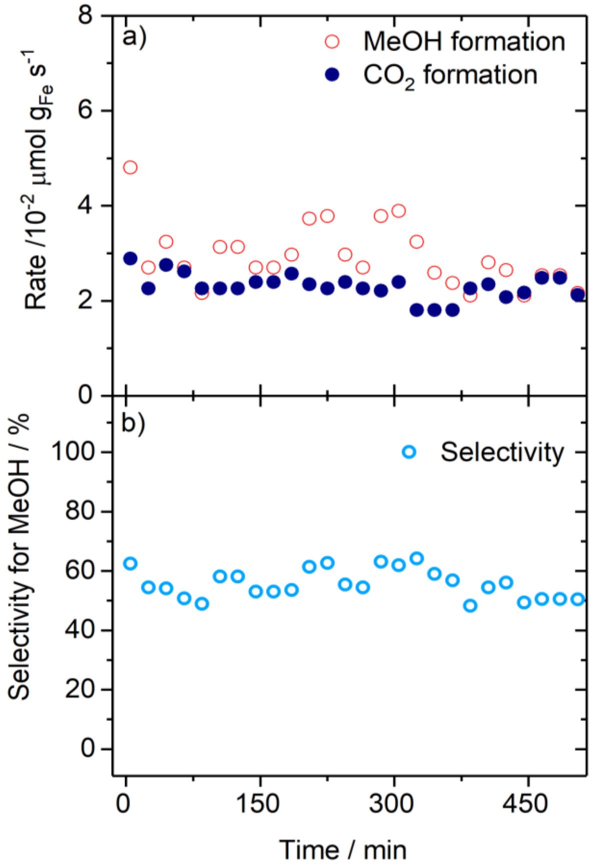

Figure S12. On-stream partial oxidation of methanol after the reactivation step of the spent catalyst. The reactivation was performed by treating the catalyst with i) 10% O_2/Ar at 250 °C for 1 h at 1bar), ii) steaming with 5% H_2O/Ar for 1 h at 1 bar, and iii) N_2 for 30 h. Then, the kinetic measurement was performed using a mixture of 10% CH_4, 5% O_2 + 0.5 % H_2O, balance Ar at 5 bar and 180 °C. **a** Rate of formation of methanol (○) and CO_2 (●) during the direct activation of CH_4 at 5 bar and 180 °C. **b** Selectivity (○) toward methanol formation ([MeOH]/{[MeOH] + [CO_2]}).

Transient kinetic isotopically labelled methane oxidation was carried out on an *operando* DRIFTS (diffuse reflectance Fourier transform infrared spectroscopy) setup comprising a gas mixer controlled by mass flow controllers (Brooks instrument), a syringe pump (Chemyx) to supply water vapor, a reaction cell (Harrick Scientific) equipped with ZnSe windows, a mass spectrometer (Hiden Analytical). All gas lines were heated to 70 °C to prevent water vapor condensation. The catalyst (50 mg) was pretreated by exposing the catalyst sequentially to 1) Ar (30 sccm), 250 °C, 1 h, 2) Ar (30 sccm), 15 min, 3) 10% O_2/Ar (30 sccm), 250 °C, 1 h, 4) Ar (30 sccm), 15 min, 5) 5% H2O/Ar (30 sccm) , 250 °C, 1 h, and 6) Ar (30 sccm) while decreasing the temperature to 180 °C. These pretreatment conditions are similar to the kinetic measurements performed on a tubular flow microreactor described in the main text. Then, the catalytic methane oxidation measurements were performed in 10% $^{13}CH_4$, 5% O_2 + 0.2 % H_2O, balance Ar (total flow 30 sccm) at 1 bar and 180 °C for 30 min. After that, the $^{13}CH_4$ feed was exchanged to $^{12}CH_4$. During these measurements, the signals at the outlet were monitored using an online mass spectrometer equipped with a Faraday cup and SEM detectors. Note that the pressure is limited by the pressure of the commercially available $^{13}CH_4$ cylinder (Cambridge Isotope Laboratories) which is 1 bar.

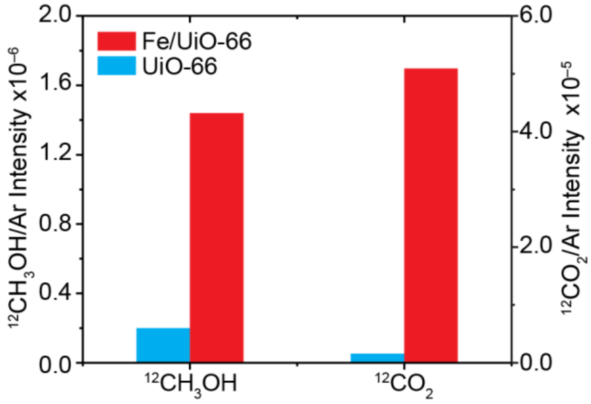

Figure S13. Steady-state partial methane oxidation activity tracked by online mass spectrometry over Fe/UiO-66 and UiO-66. The experiments were carried out similarly to the transient kinetic isotopically labeled methane oxidation described above except only $^{12}CH_4$ was used as a methane source. The Ar gas normalized signals were subtracted from the signals obtained without any catalyst.

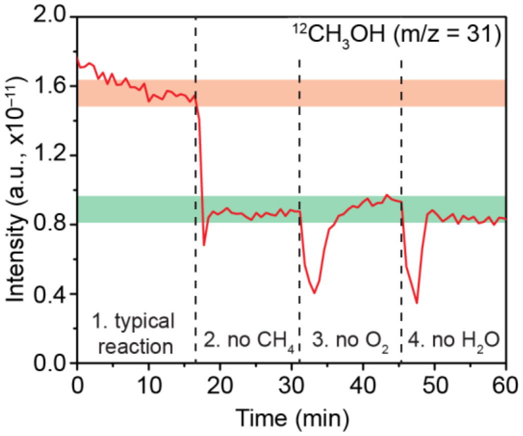

Figure S14. Verification of methane oxidation activity at 1 bar and 180 °C over Fe/UiO-66 using different gas compositions: 1) 10% CH_4 + 5% O_2 + 0.2 % H_2O, balance Ar (total flow 30 sccm), 2) 5% O_2 + 0.2 % H_2O, balance Ar (total flow 30 sccm), 3) 10% CH_4 + 0.2 % H_2O, balance Ar (total flow 30 sccm), 4) 10% CH_4 + 5% O_2, balance Ar (total flow 30 sccm). Online mass spectroscopy signals of $^{12}CH_3OH$. The catalyst pretreatment and the catalytic experiments were carried out following the procedure described in Figure S15.

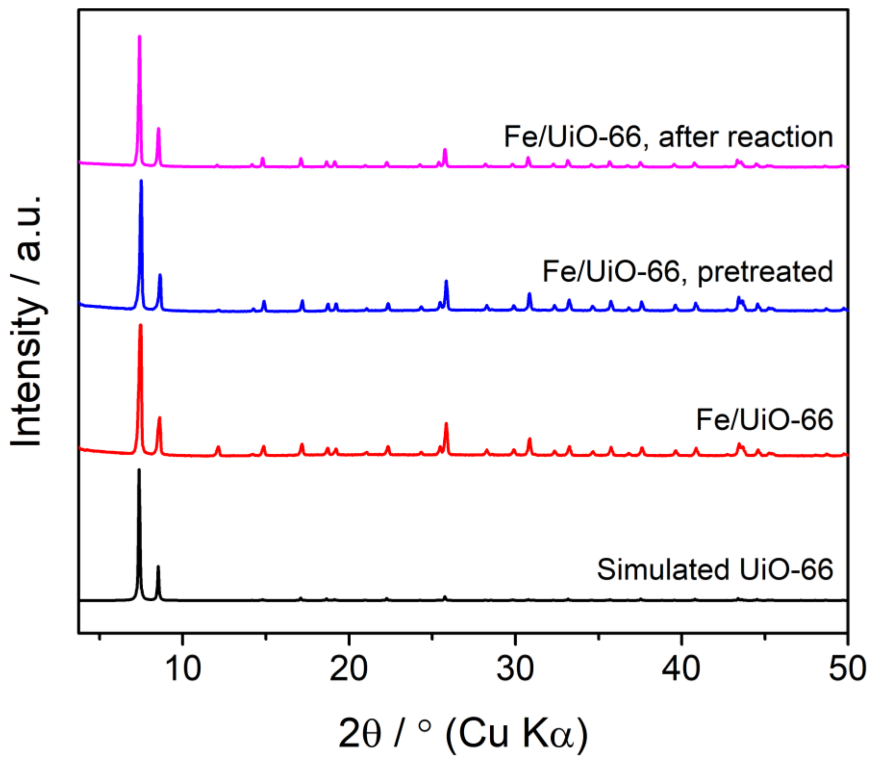

Figure S15. PXRD patterns of Fe/UiO-66 (red), pretreated Fe/UiO-66 (blue), Fe/UiO-66 after the reaction, and simulated UiO-66 (black).

Section S5. Analysis of FeO_x species using high-resolution transmission electron microscopy analysis

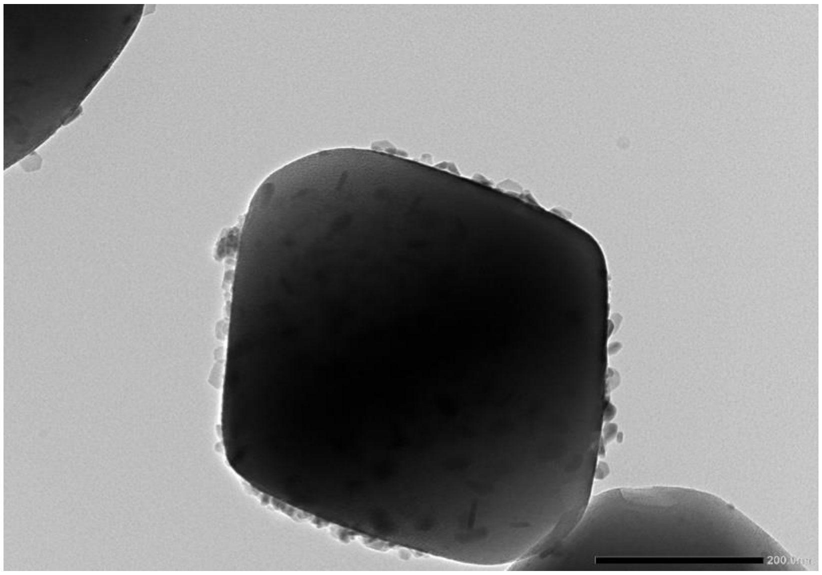

Figure S16. Overview TEM image of pre-treated Fe/UiO-66.

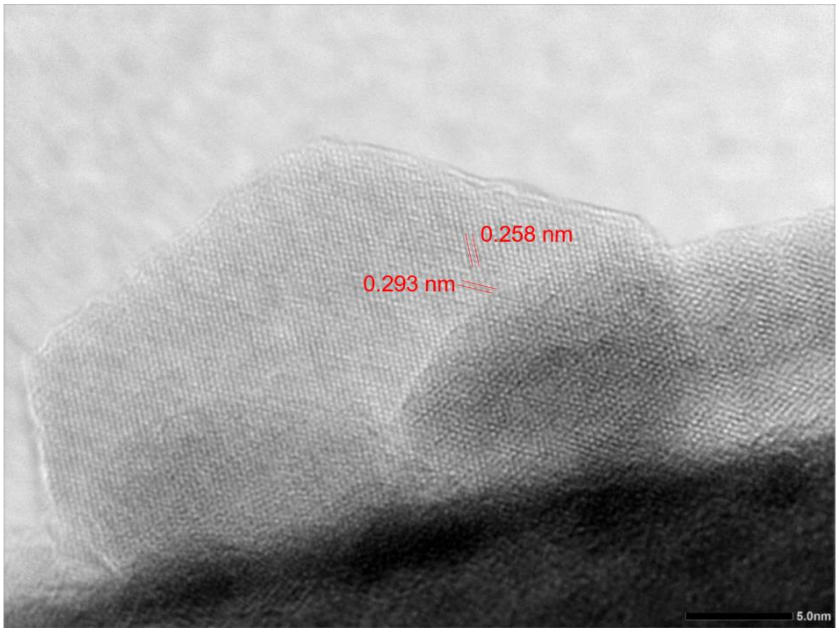

Figure S17. HR-TEM image of pre-treated Fe/UiO-66 focusing at 'FeO_x' nanoparticle. The d-spacing of 0.258 and 0.293 nm corresponds to the d-spacing of (311) and (220) planes of γ-Fe_2O_3, respectively.[17]

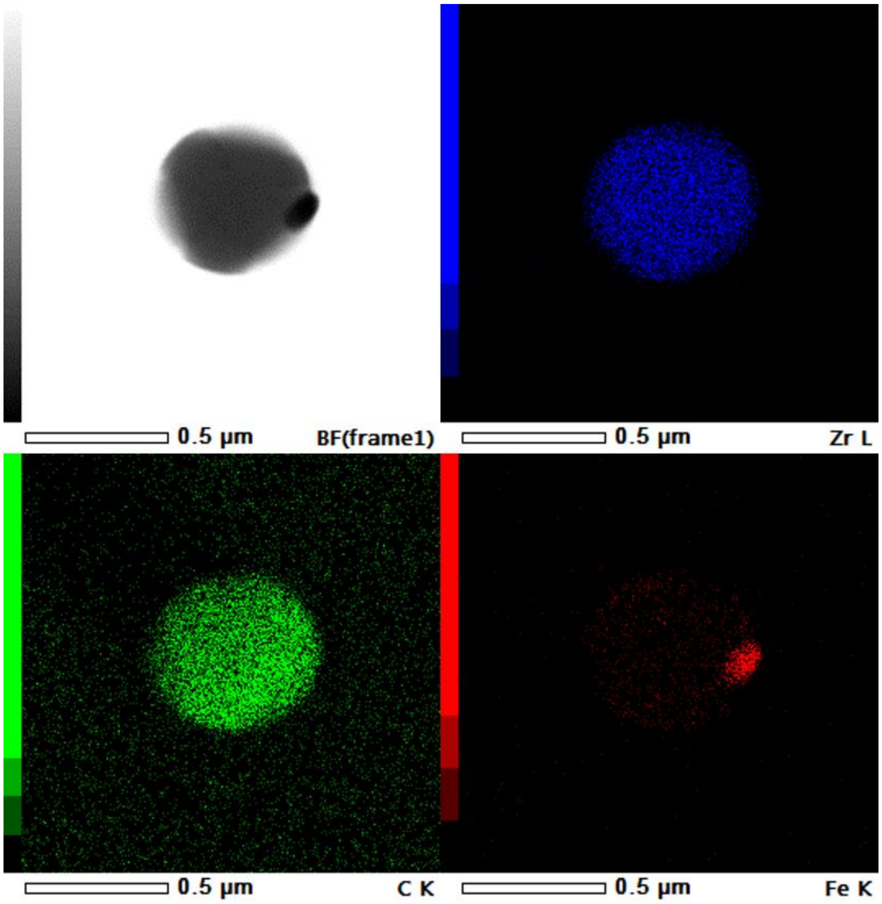

Figure S18. Elemental mapping of pretreated Fe/UiO-66 using STEM-EDS.

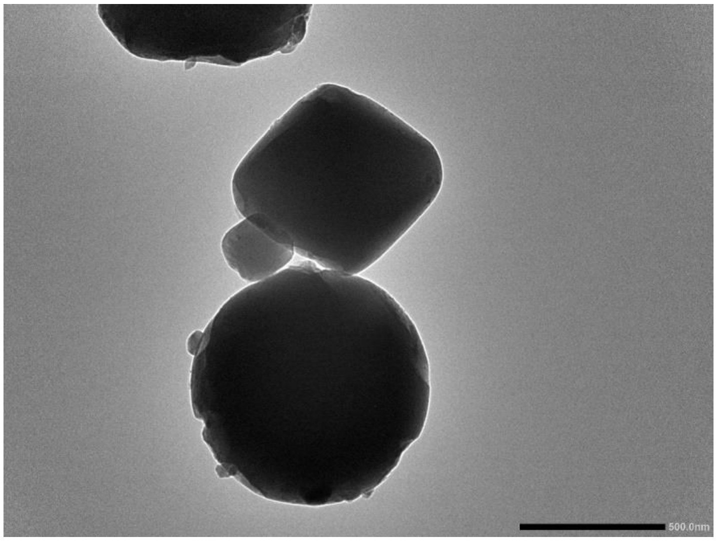

Figure S19. TEM image of spent Fe/UiO-66.

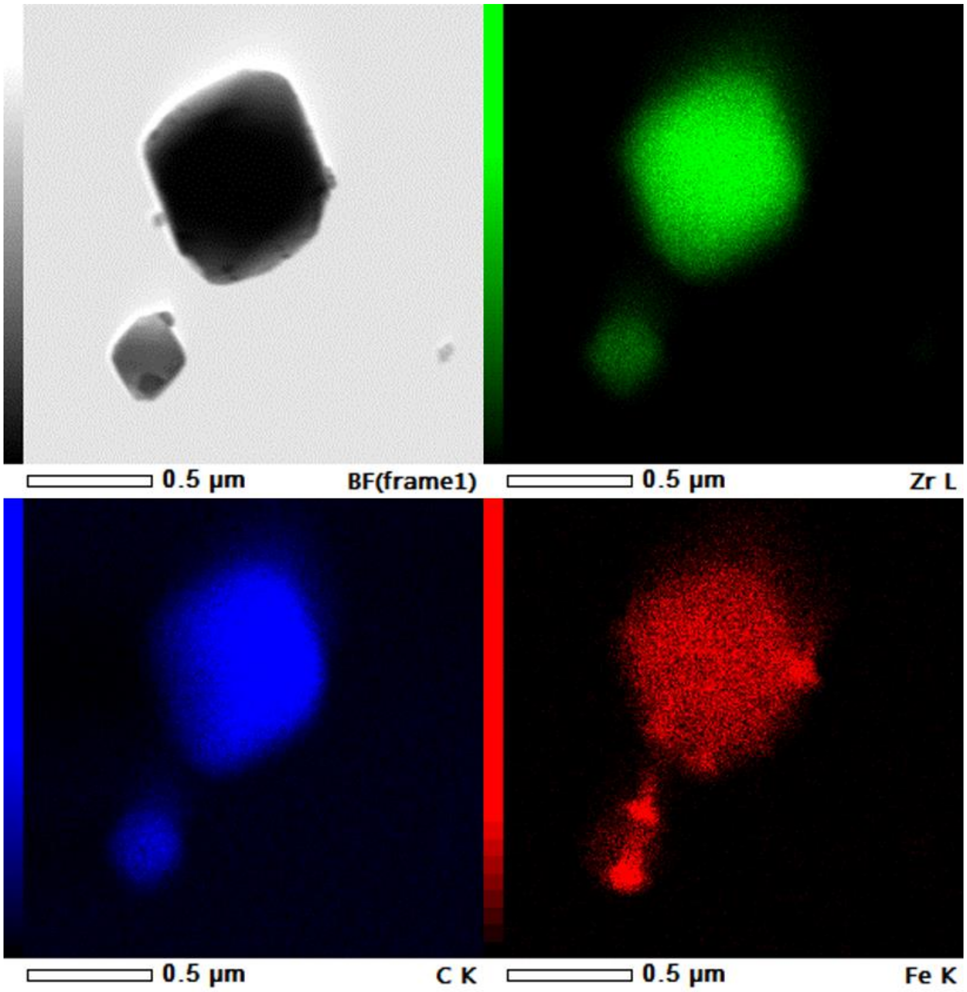

Figure S20. Elemental mapping of spent Fe/UiO-66 using STEM-EDS.

Section S6. Mössbauer spectroscopy and EPR spectroscopy

Zero-field ^{57}Fe Mössbauer spectra were recorded in a constant acceleration spectrometer (SEE Co., Minneapolis, MN) which utilized a cobalt-57 gamma source embedded in a Rh matrix. The powder Fe/UiO-66 catalyst was placed in a nylon washer (80 mg/cm^2) and sealed with Kapton® tape prior to introduction into the spectrometer. The sample was then placed in a cryostat (Janis Research Co., Willmington, MA) and cooled down by a helium closed cycle refrigerator. The gamma source was kept at room temperature. Isomer shifts are reported relative to α-iron (30 µm foil) at 295 K. EPR spectrum was recorded at 140 K using a Bruker spectrometer (Elexys 500) at the X-band frequency of 9.40 GHz.

Table S4. Fitting parameters for Mössbauer spectra of as-synthesized Fe/UiO-66

Temperature (K)	80	5
**Site 1**		
δ (mm/s)	0.474(1)	0.513(4)
ΔE_Q (mm/s)	1.04(1)	0.863(7)
Γ (mm/s)	0.530(7)	0.84(1)
Relative Area (%)	50(2)	45.9(4)
**Site 2**		
δ (mm/s)	0.492(1)	0.453(7)
ΔE_Q (mm/s)	0.571(7)	0.05(1)
Γ (mm/s)	0.416(7)	0.99(2)
H_{int} (mT)	–	47.86(6)
Relative Area (%)	50(2)	54.1(7)

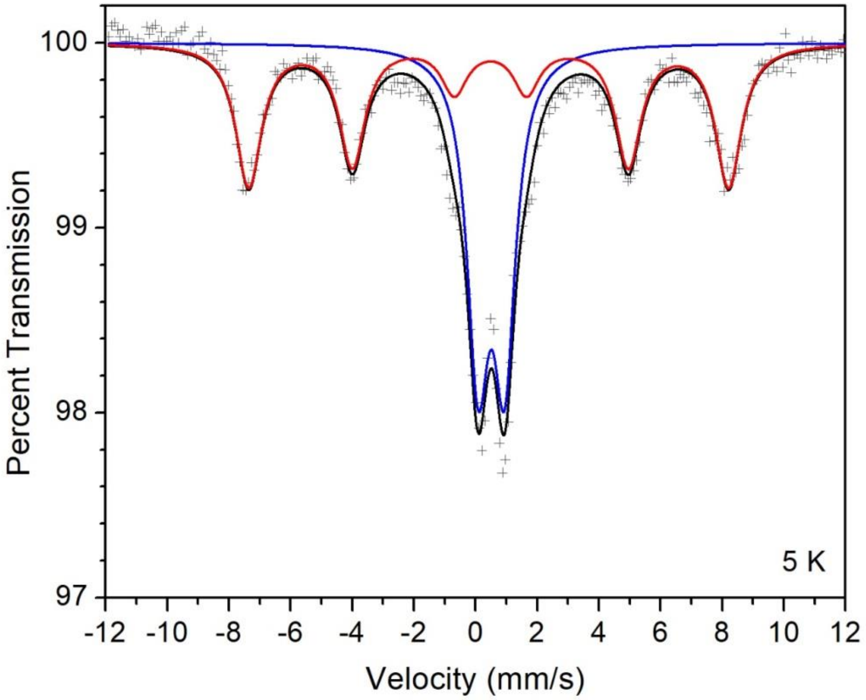

Figure S21. Mössbauer spectrum of the as-synthesized catalyst collected at 5 K plotted as gray crosses. The fit to the spectrum is shown in black solid line, and the contributions from the first and second sites shown in blue and red lines, respectively.

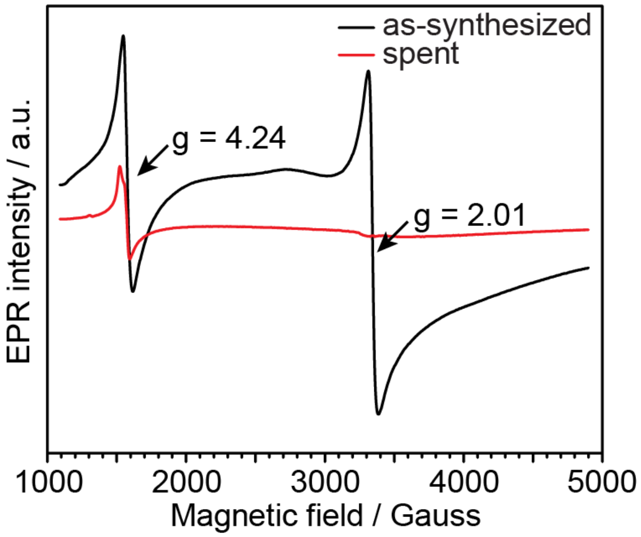

Figure S22. EPR spectra of the as-synthesized and spent catalyst collected at 140 K.

Section S7. X-ray Absorption Spectroscopy (XAS)

Fe K-edge X-ray absorption spectroscopy data were collected at the BL 5.2 SUT-NANOTEC-SLRI, a bending-magnet beamline with the storage ring operating at a current of 80–150 mA and 1.2 GeV at the Synchrotron Light Research Institute (SLRI), Thailand. A Ge (220) double crystal monochromator with an energy resolution ($\Delta E/E$) of 2×10^{-4} was used to scan the synchrotron X-ray beam. Energy calibrations were carried out using metal Fe foil (7112 eV). The powder of the as-synthesized, pretreated and the spent Fe/UiO-66 catalyst samples were loaded into plastic frames sealed with thin polypropylene film and Kapton tape. All data were collected at room temperature (23 °C) in fluorescence mode with the step size of 0.02 eV in the X-ray absorption near edge structure (XANES) region. In the extended X-ray absorption fine structure (EXAFS) region, the data were recorded up to 14k. The data reduction and EXAFS curve fitting were performed using the Athena and Artemis software, respectively, which are included in an IFEFFIT package. All the fits were performed in k and R spaces over 2.5–11.5 Å^{-1} and 1–3.8 Å^{-1}, respectively. All parameters were freely optimized during the EXAFS data fitting where the cif files of ferrihydrite and Fe_2O_3 were employed as models.

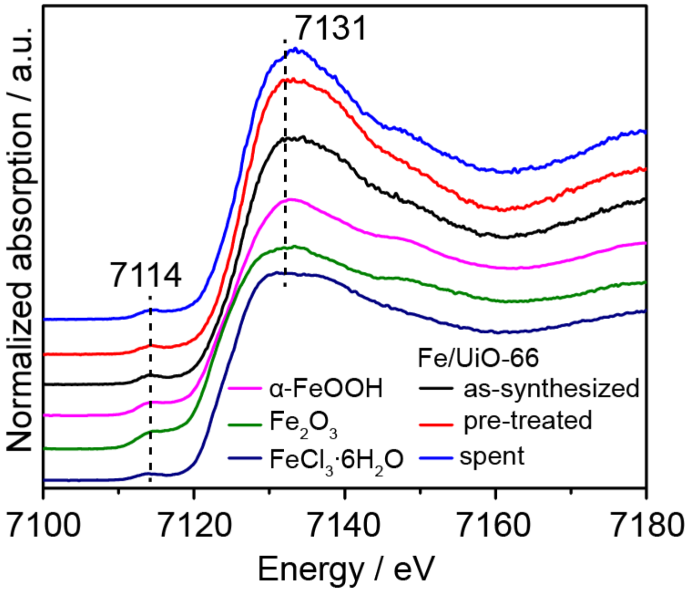

Figure S23. Fe K-edge XANES spectra of Fe/UiO-66 in as-synthesized, pre-treated and spent states overlaid the reference spectra including α-FeOOH, Fe_2O_3, $FeCl_3 \cdot 6H_2O$.

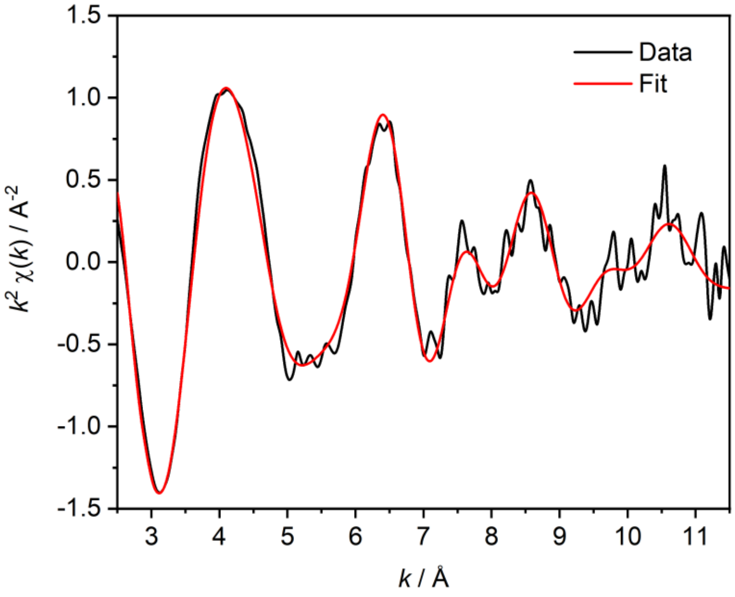

Figure S24. The k^2-weighted Fe K-edge EXAFS spectra of as-synthesized Fe/UiO-66 (black line) and best fit 1(red line) in *k*-space.

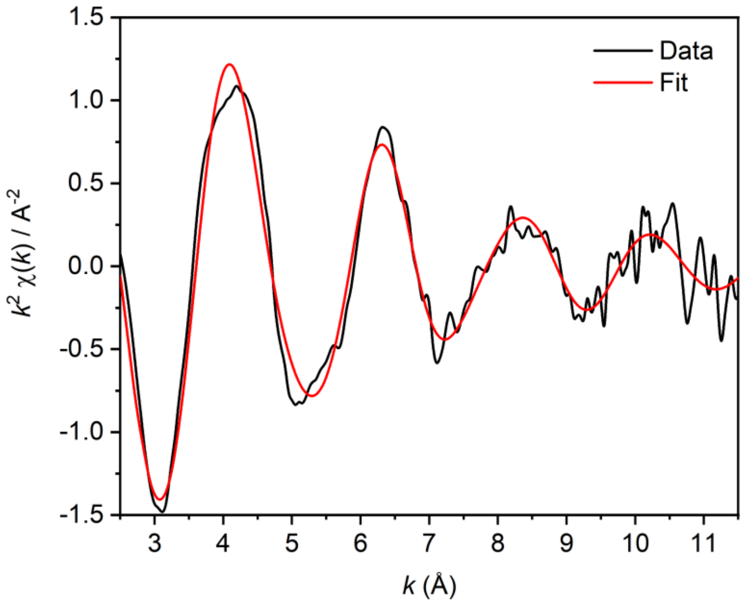

Figure S25. The k^2-weighted Fe K-edge EXAFS spectra of pre-treated Fe/UiO-66 (black line) and best fit (red line) in *k*-space.

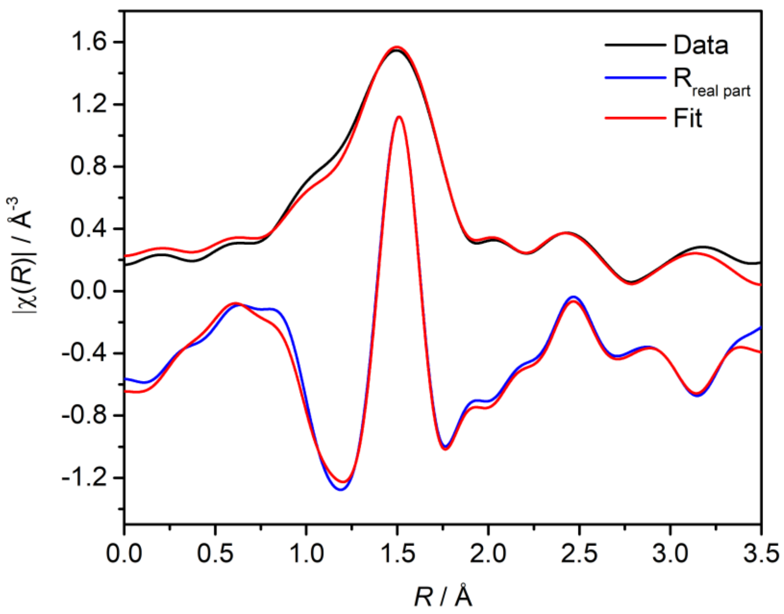

Figure S26. Fourier transformed Fe K-edge EXAFS spectrum of the pre-treated Fe/UiO-66 catalyst without phase correction.

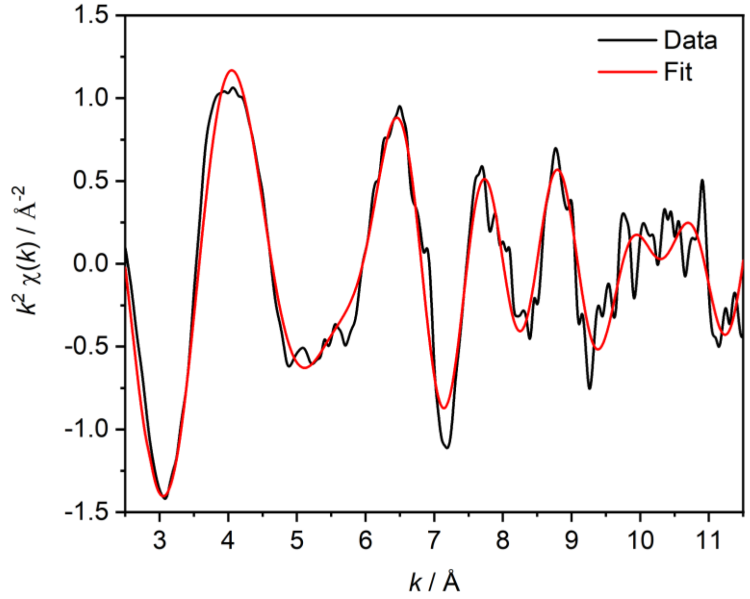

Figure S27. The k^2-weighted Fe K-edge EXAFS spectra of spent Fe/UiO-66 (black line) and best fit (red line) in *k*-space.

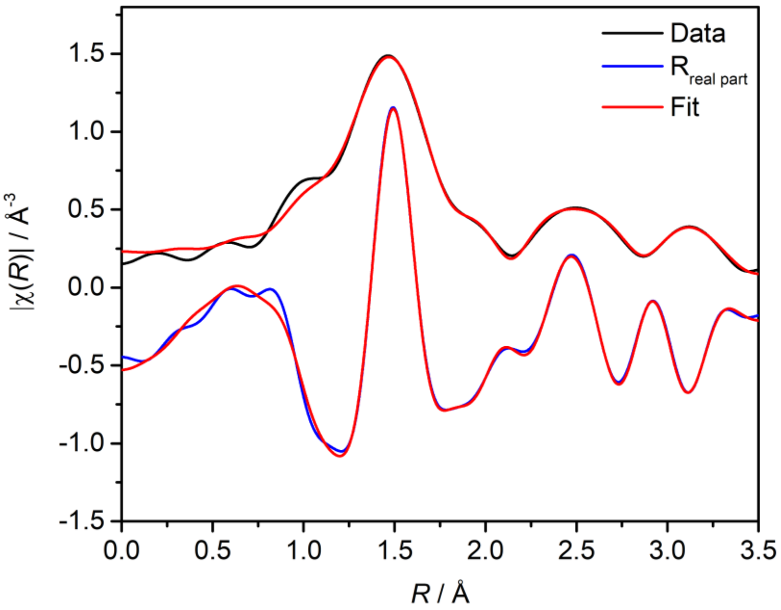

Figure S28. Fourier transformed Fe K-edge EXAFS spectrum of the spent Fe/UiO-66 catalyst without phase correction.

To inspect for possible presence of ultrasmall Fe_2O_3 cluster instead of isolated Fe sites, we have fitted the EXAFS data using the Fe_2O_3 model alone and allowed all the parameters to be refined including the CN which is the parameter directly related to the cluster size. The best fit we obtained gave negative σ^2 values which are unacceptable (see Figure S30, S31 below and Table S5). Therefore, we can rule out the possibility of having a very small cluster of iron oxide instead of isolated Fe sites in the sample.

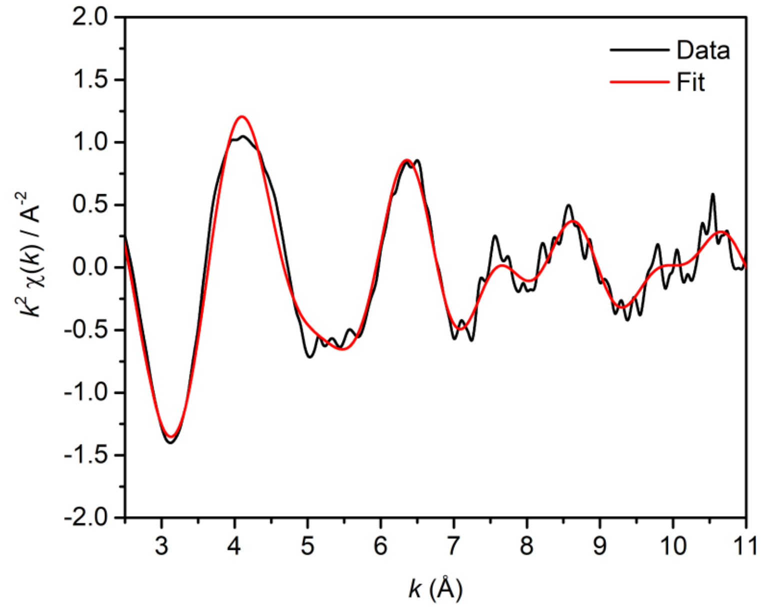

Figure S29. The k^2-weighted Fe K-edge EXAFS spectra of as-synthesized Fe/UiO-66 (black line) and best fit (red line) in k-space using γ-Fe_2O_3 as a model.

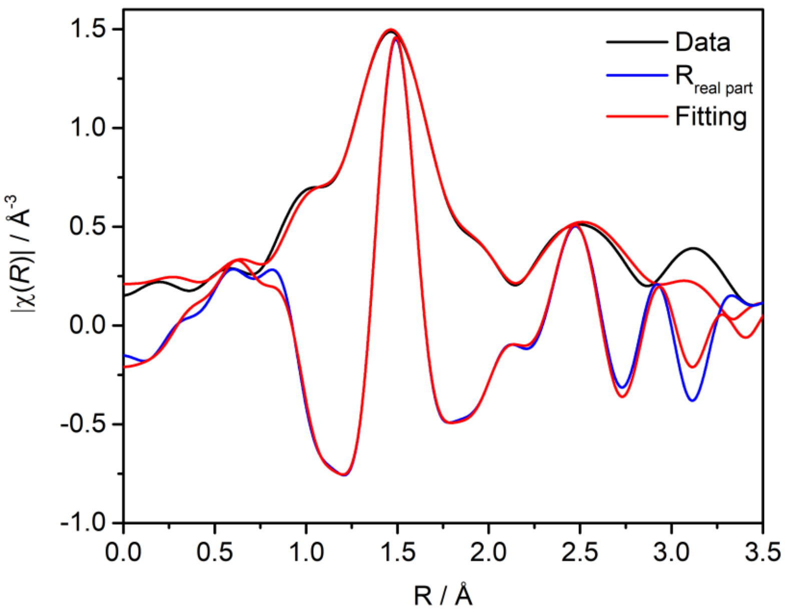

Figure S30. Fourier transformed Fe K-edge EXAFS spectrum of the as-synthesized Fe/UiO-66 catalyst using γ-Fe_2O_3 as a model without phase correction.

Table S5. Fe K-edge EXAFS fitting result of the as-synthesized Fe/UiO-66 using using γ-Fe_2O_3 as a model.

Ab-Sc pair[a]	CN[b]	R[c]	DWF[d]	R-factor[e]
Fe–O1	1.32 ± 0.52	1.86 ± 0.08	–0.0075 ± 0.0172	0.02
Fe–O2	0.66 ± 0.26	1.93 ± 0.10	–0.0170 ± 0.0145	
Fe–O3	0.66 ± 0.26	2.07 ± 0.08	–0.0160 ± 0.0139	
Fe–O4	0.66 ± 0.26	2.23 ± 0.09	–0.0145 ± 0.0151	
Fe···Fe1	0.66 ± 0.26	2.91 ± 0.07	–0.0036 ± 0.0083	
Fe···Fe2	0.66 ± 0.26	3.06 ± 0.07	–0.0050 ± 0.0069	

[a]Ab = absorber; Sc = scatterer. [b] Coordination number. [c] Distance (Å). [d] Debye-Waller factor ($Å^2$). [e]A measure of mean square sum of the misfit at each data point. Fit range: 2.5 < k < 11 $Å^{-1}$; 1 < R < 3.8 Å; Fit window: Hanning.

Section S8. X-ray photoelectron spectroscopy

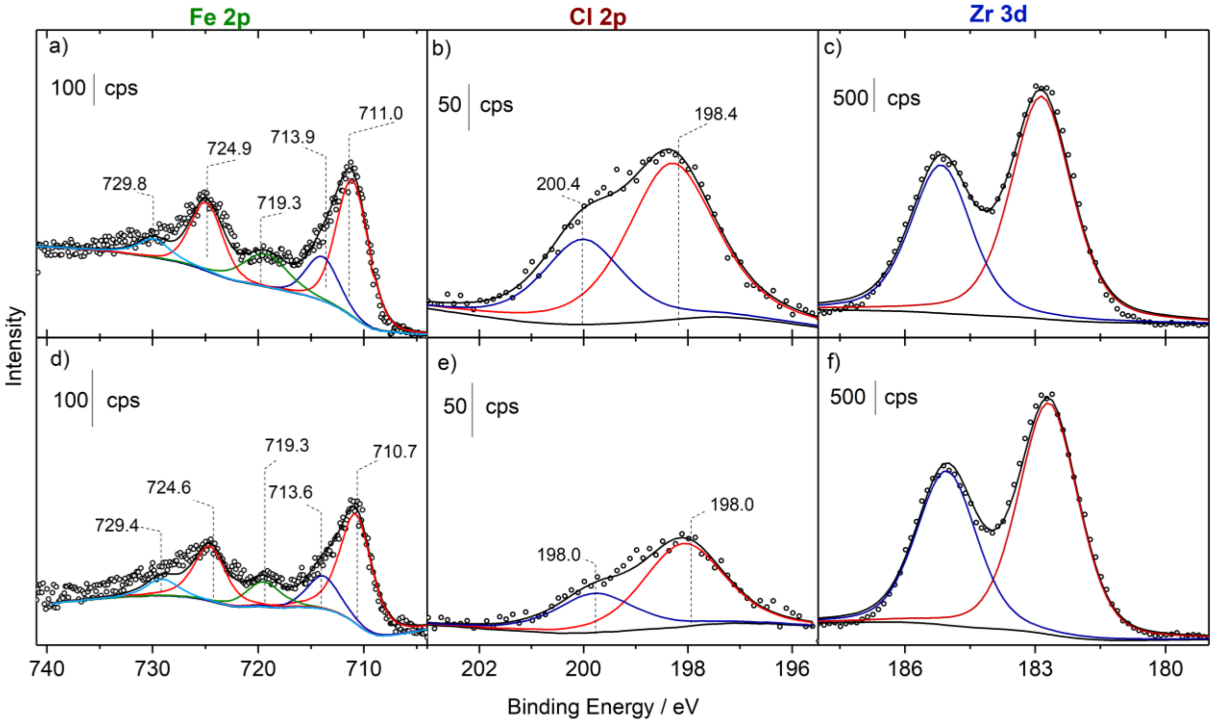

Figure S31. XPS spectra in the Fe 2p region (a, d) Cl2p (b,e) and Zr 3d (c, f) regions of the fresh sample after treatment (upper panels: 1,b,c) and after methane activation reaction (d, e, f) in the same gas mixture described in the kinetic measurements (10% CH_4, 5% O_2 + 0.5 % H_2O, balance Ar) at 5 bar and 180 °C. c). All binding energies were calibrated using C1s native carbon as reference (284.8 eV)

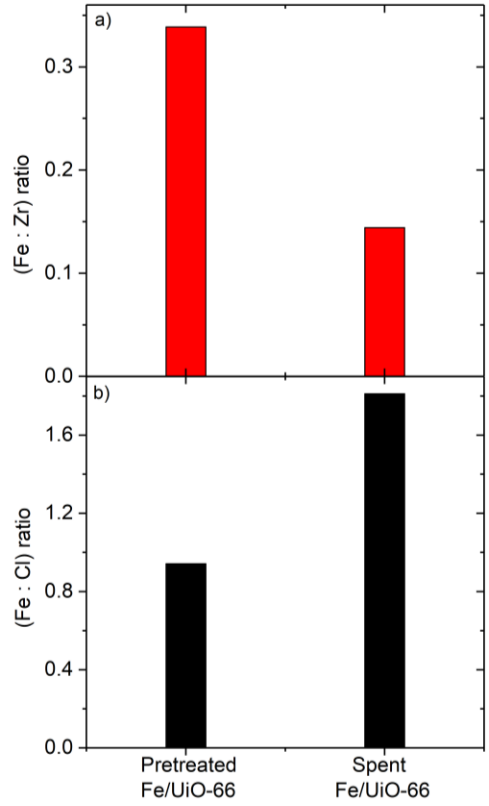

Figure S32. Atomic surface ratios of Fe:Zr (a) and of Fe : Cl (b) obtained from XPS measurements (see Figure S25) after pretreatment (i: 10% O_2/Ar at 250°C for 1 h at 1 bar; ii) steaming by 5 %H_2O /Ar for 1 h at 1 bar, and iii) purging with N_2 for 15 min) and after a subsequent reaction in standard gas mixture (10% CH_4, 5% O_2 + 0.5 % H_2O, balance Ar) at 5 bar and 180–190 °C.

Section S9. Density functional theory calculations

All calculations were performed using Gaussian 09 program suite.[18] The cluster model was formed by extracting one zirconium node from the crystal structure of UiO-66.[2] The octahedral Fe^{3+} in the zirconium node of UiO-66 was then constructed by removing one 1,4-benzenedicarboxylate (BDC) linker and placing Fe atom with four equatorial hydroxyl ligands and two axial H_2O ligands at the defect site, which we denoted as Fe/UiO-66, Fe^{3+}-$Zr_6O_4(OH)_8(H_2O)_2$(benzenecarboxylate)$_{11}$ **(I)** as shown in Fig 5a in the main text.

Geometry optimizations were carried out using the M06-L local density functional. The 6-31G* basis set was used for C, N, O, and H atoms and the Stuttgart-Dresden (SDD) pseudopotentials basis set was used for Fe and Zr atoms. During geometry optimizations, each C atom of carboxylates was constrained while the remaining atoms are fully relaxed.

Table S6 Electronic energies of Fe/UiO-66 with varying spin state.

System	Spin multiplicity	Relative energy (kcal/mol)*
Fe/UiO-66 **(I)**	Doublet	19.9
	Quartet	7.7
	Sextet	0.0

* Relative energy compared with the most stable spin state

XYZ coordinates

Fe/UiO-66 (sextet spin state)

C	-2.622800	-2.941300	4.423900
C	2.756600	2.939500	-4.411800
C	-4.937500	3.052600	-1.008500
C	5.071300	-3.054300	1.020600
C	-5.028700	-2.896100	-1.017200
C	5.162600	2.894500	1.029500
C	2.665300	-3.009300	-4.420500
C	-2.531500	3.007700	4.432700
C	-0.024500	-5.949700	-0.002700

C	0.158200	5.948000	0.014900
C	2.472900	-0.045900	5.447300
Zr	0.756100	-1.785300	1.612500
Zr	-0.643100	1.792200	-1.635000
Zr	-2.227200	0.035800	1.008000
Zr	2.352200	-0.035500	-1.028300
Zr	-0.695100	-1.768000	-1.639600
Zr	0.812500	1.758200	1.616500
O	-0.732300	-2.485400	3.118500
O	0.923600	2.592000	-3.011900
O	-2.726100	2.543400	-1.529700
O	2.820400	-2.601100	1.467200
O	-3.784200	-1.319000	0.177000
O	3.894400	1.361600	-0.208900
O	2.758000	-1.432800	-2.694100
O	-2.590900	1.388100	2.744400
O	-0.462500	-3.902400	-1.048700
O	0.634800	3.888400	1.010600
O	1.622100	1.103400	3.591700
O	-2.634000	-1.311800	2.743100
O	2.795200	1.359400	-2.687600
O	3.849700	-1.480500	-0.212100
O	-3.735700	1.432300	0.171300
O	1.585900	-1.162200	3.588600
O	-0.314000	3.916500	-1.046600
O	0.509100	-3.907300	0.998300
O	-0.653500	2.498700	3.127400
O	0.866300	-2.641800	-2.982900
O	-2.818600	-2.409200	-1.563500

O	2.899700	2.508500	1.473300
C	7.577100	4.179600	1.591300
C	-7.401900	-4.300300	-1.467100
C	-3.782400	4.475400	6.451800
C	3.818800	-4.297100	-6.615300
C	-1.943000	-2.183400	3.353400
C	2.112300	2.251900	-3.278400
C	-3.711000	2.274100	-0.774500
C	3.818100	-2.329000	0.730900
C	-3.784100	-2.145300	-0.782800
C	3.888800	2.207900	0.736500
C	2.052500	-2.316700	-3.272700
C	-1.873400	2.234700	3.359600
C	0.017000	-4.476100	-0.023100
C	0.166900	4.473700	-0.013100
C	1.839000	-0.033800	4.112400
C	3.970500	4.223200	-6.575700
C	-3.915000	-4.379200	6.438500
C	7.446700	-4.412400	1.578200
C	-7.280600	4.508100	-1.457600
C	0.129700	8.740200	0.073600
C	-0.111900	-8.740900	0.040800
C	3.684600	-0.068600	7.962500
C	6.438100	4.479800	2.336800
H	6.488900	5.215200	3.137900
C	-6.325800	-4.453000	-2.340000
H	-6.409200	-5.117500	-3.198100
C	-2.441900	4.709200	6.148800
H	-1.882300	5.463700	6.699000

C	2.531600	-4.639300	-6.203800
H	1.975500	-5.409000	-6.736000
C	4.531300	-3.313900	-5.930800
H	5.537000	-3.047100	-6.250900
C	-4.498300	3.508200	5.748100
H	-5.544700	3.324400	5.985500
C	7.511100	3.239100	0.564600
H	8.400200	3.005100	-0.018300
C	-7.295900	-3.444100	-0.371800
H	-8.136500	-3.323600	0.309300
C	-1.940600	-3.942600	5.124600
H	-0.903300	-4.144100	4.867800
C	2.074900	3.942200	-5.111900
H	1.070400	4.210100	-4.792300
C	-4.978600	4.038500	-2.001800
H	-4.084600	4.226400	-2.591700
C	5.116700	-4.005000	2.047100
H	4.211500	-4.200500	2.617100
C	-6.118000	-2.741000	-0.150400
H	-6.017700	-2.063500	0.694400
C	6.309800	2.599600	0.283500
H	6.237500	1.863900	-0.514200
C	3.958800	-2.673800	-4.838200
H	4.499100	-1.904900	-4.290700
C	-3.876700	2.777000	4.743100
H	-4.417400	2.017200	4.183700
C	-0.577200	-6.656200	-1.077500
H	-0.967800	-6.096200	-1.924000
C	0.675900	6.636700	1.118200

H	1.087500	6.062300	1.944800
C	2.796200	1.157000	6.085700
H	2.564900	2.092600	5.582000
C	-3.957400	-2.665300	4.743400
H	-4.472900	-1.881500	4.193100
C	4.053600	2.588700	-4.805200
H	4.572700	1.810400	-4.250700
C	6.226300	-2.792200	0.274500
H	6.175900	-2.053000	-0.521500
C	-6.084800	2.800200	-0.246300
H	-6.042000	2.025600	0.515700
C	2.760100	-1.260300	6.081300
H	2.500700	-2.186800	5.574400
C	-0.371900	6.672000	-1.059600
H	-0.768500	6.124700	-1.911600
C	0.486400	-6.655400	1.093000
H	0.916600	-6.094500	1.919400
C	-1.817900	3.978800	5.144600
H	-0.772600	4.145200	4.894600
C	1.956600	-3.999000	-5.112300
H	0.954900	-4.255300	-4.774600
C	-5.143500	-3.756700	-2.116500
H	-4.296700	-3.861600	-2.790700
C	5.235700	3.841000	2.058100
H	4.336300	4.062300	2.628000
C	4.656200	3.227000	-5.882600
H	5.664600	2.948800	-6.183900
C	-4.599600	-3.381600	5.746100
H	-5.637300	-3.161900	5.991100

C	7.408100	-3.468000	0.553600
H	8.303500	-3.259400	-0.029400
C	-7.249000	3.525300	-0.469200
H	-8.136900	3.323700	0.127600
C	2.680000	4.580500	-6.188000
H	2.145100	5.360400	-6.727100
C	-2.585400	-4.658300	6.126400
H	-2.050300	-5.436500	6.667800
C	-6.144100	4.762900	-2.223200
H	-6.167400	5.528600	-2.996600
C	6.299600	-4.680100	2.323700
H	6.328900	-5.418500	3.123000
C	-0.386700	8.061300	-1.028900
H	-0.801400	8.618500	-1.867200
C	0.442500	-8.044200	1.113400
H	0.842200	-8.587300	1.968000
C	3.363700	-1.270200	7.333000
H	3.585000	-2.217600	7.821500
C	3.399600	1.144400	7.337500
H	3.649100	2.082900	7.829400
C	-0.621400	-8.045000	-1.054300
H	-1.053500	-8.588500	-1.892700
C	0.661300	8.026000	1.146100
H	1.066200	8.555800	2.006700
O	-1.264400	-1.978100	0.564100
O	0.472000	-0.007200	-2.470700
O	-1.196700	2.017900	0.569900
O	2.173100	-0.034700	1.253700
O	-1.474500	-1.408000	-3.696900

O	-1.521500	1.445000	-3.663900
H	-1.791000	-2.753200	0.798100
H	0.626900	-0.025000	-3.423900
H	-1.703000	2.807400	0.800300
H	3.001200	-0.048100	1.750600
O	-0.292700	0.003100	1.769500
O	1.033700	1.447200	-0.437100
O	0.989300	-1.478400	-0.437700
O	-1.521300	0.025000	-0.963500
H	0.118300	9.828800	0.096700
H	-8.193300	5.075800	-1.632300
H	-8.326400	-4.848600	-1.641800
H	-0.146100	-9.829200	0.058000
H	8.518700	4.680700	1.810800
H	8.373100	-4.941700	1.796000
H	4.157600	-0.077400	8.943300
H	4.267700	-4.798600	-7.471300
H	4.443200	4.723400	-7.419600
H	-4.270100	5.048200	7.239100
H	-4.418700	-4.940300	7.224100
H	-2.111900	2.179000	-3.914400
H	-1.954500	-2.175000	-4.055300
Fe	-2.701300	0.005300	-4.325300
O	-3.704100	-1.382600	-4.980900
O	-3.759200	1.364500	-5.021800
H	-4.415900	-1.023900	-5.531800
H	-3.770900	1.281900	-5.987700
O	-1.101700	0.148800	-6.073000
H	-0.702300	0.863600	-5.542400

H	-0.678200	-0.660400	-5.738300
O	-4.357400	0.171900	-2.675100
H	-4.771600	0.832300	-3.263300
H	-4.758500	-0.667600	-2.958400

Section S10. In situ diffuse reflectance UV-Vis spectroscopy

UV–Vis diffuse reflectance spectra were recorded by an Ava Spec-2048 spectrometer equipped with a FCR-7UV400C-2 reflection probe (Avantes, Apeldoorn, Netherlands) in the energy range 1.5–7 eV. The samples were placed in a commercial in-situ reaction chamber (HVC-MRA-5, Harrick, Pleasantville, NY). Samples was treated as follows. First we heated the sample under a flow of Ar (50 mL min^{-1}) from room temperature to 250 °C. The sample was dried for 1 h to get rid of any adsorbed solvent molecules left from the synthesis. After that, the sample was exposed to 10% O_2/Ar at 250 °C for 1 h and eventually was purged in Ar for 15 min. After that we exposed the to a steam mixture (5% H_2O/Ar) for 1 at 250 °C. Then, the sample was cooled down to 180 °C and DR-UV-vis spectra were collected during methane oxidation in the gas mixture of 10% CH_4 + 5% O_2 +2% H_2O balanced in Ar.

Section S11. Identification of probable reactive intermediates using in situ DRIFTS.

In situ DRIFT spectra were recorded on a Magna6700 spectrometer (Thermo-Fisher) equipped withan MCT narrow-bandgap detector in a commercial reaction cell (HV-DR2,Harrick), which is identical to the cell use din DR-UV-vis. The cup of the reaction cell (see Fig S34a) was filled by about 50 mg of the catalyst and pretreatment was carred out under continuous flow of pretreatment and reaction gases with a composition identical to that used in the kinetic measurements described in experimental part.

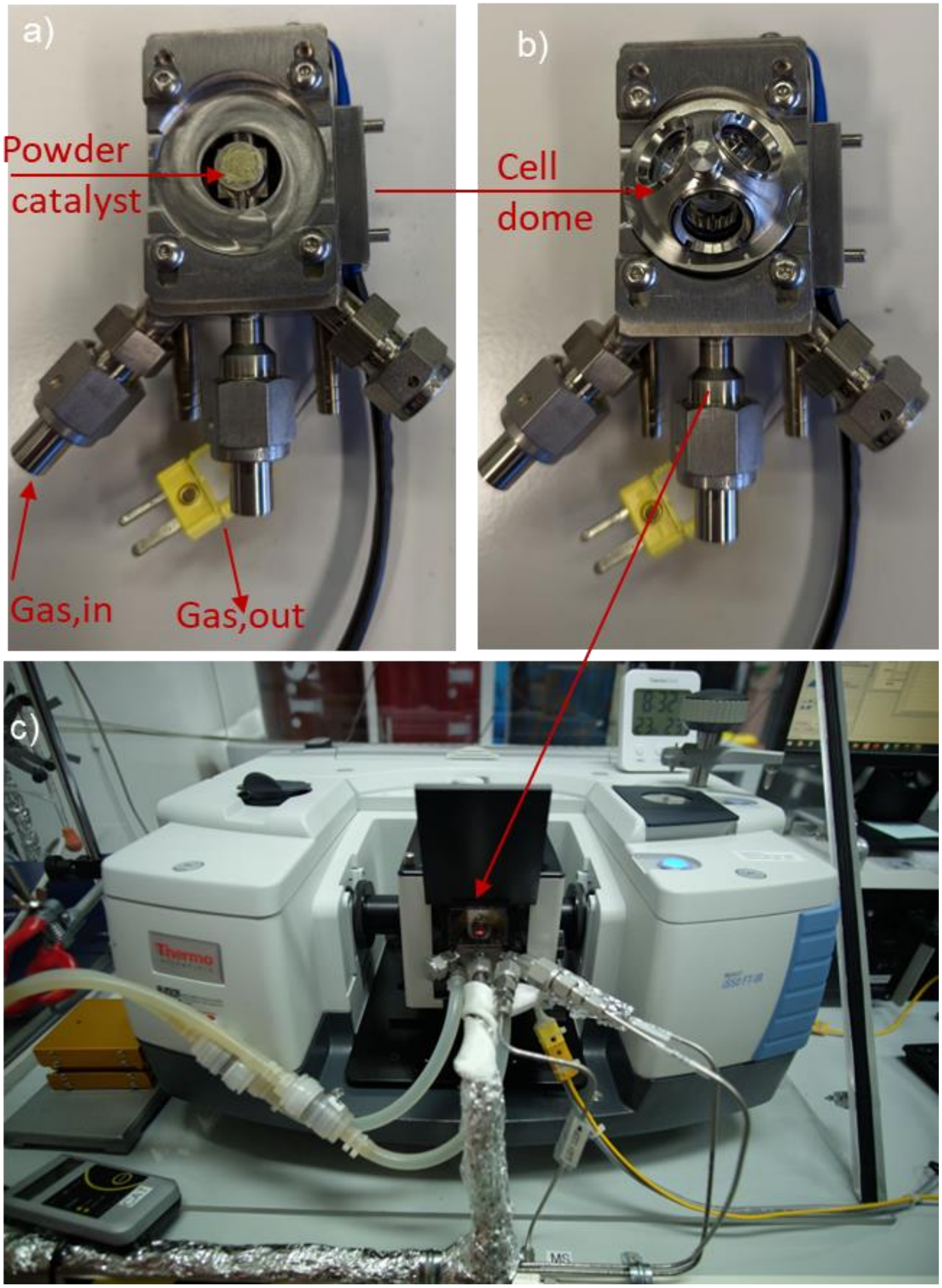

Figure S34. Reaction steup used in the diffuse reflectance FTIR measurmenets. (a) Harricks cell filled with the powder catalyst, (b) reaction cell covered with the dome. (c) Reaction cell built inside the Praying-Mantis suited into the photon path in the DRIFTS spectrometer.

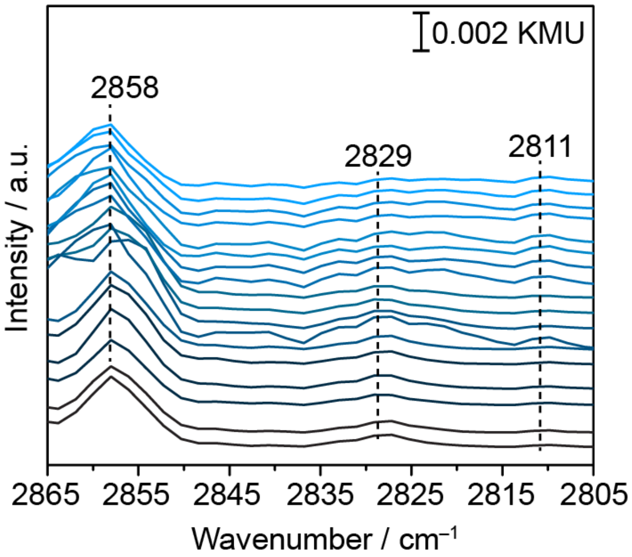

Figure S33. A sequence of in situ DRIFTS spectra in the C–H stretch region, characteristic peaks for surface methoxy species, during the direct activation of CH_4 (10% CH_4, 5% O_2 + 0.2 % H_2O, balance Ar) at 5 bar and 180 °C.

Section S11. References

1. Na, K.; Choi, K. M.; Yaghi, O. M.; Somorjai, G. A., Metal Nanocrystals Embedded in Single Nanocrystals of MOFs Give Unusual Selectivity as Heterogeneous Catalysts. *Nano Lett.* **2014,** *14* (10), 5979-5983.
2. Trickett, C. A.; Gagnon, K. J.; Lee, S.; Gándara, F.; Bürgi, H. B.; Yaghi, O. M., Definitive Molecular Level Characterization of Defects in UiO-66 Crystals. *Angew. Chem. Int. Ed.* **2015,** *54* (38), 11162-11167.
3. Cavka, J. H.; Jakobsen, S.; Olsbye, U.; Guillou, N.; Lamberti, C.; Bordiga, S.; Lillerud, K. P., A New Zirconium Inorganic Building Brick Forming Metal Organic Frameworks with Exceptional Stability. *J. Am. Chem. Soc.* **2008,** *130* (42), 13850-13851.
4. *APEX3, SAINT and SADABS*, Bruker AXS Inc.: Madison, Wisconsin, USA, 2016.
5. Sheldrick, G. M., SHELXT–Integrated space-group and crystal-structure determination. *Acta Crystallographica Section A: Foundations and Advances* **2015,** *71* (1), 3-8.
6. Sheldrick, G. M., Crystal structure refinement with SHELXL. *Acta Crystallographica Section C: Structural Chemistry* **2015,** *71* (1), 3-8.
7. Dolomanov, O. V.; Bourhis, L. J.; Gildea, R. J.; Howard, J. A.; Puschmann, H., OLEX2: a complete structure solution, refinement and analysis program. *Journal of applied crystallography* **2009,** *42* (2), 339-341.
8. Narsimhan, K.; Iyoki, K.; Dinh, K.; Román-Leshkov, Y., Catalytic Oxidation of Methane Into Methanol Over Copper-Exchanged Zeolites With Oxygen at Low Temperature. *ACS Cent. Sci.* **2016,** *2* (6), 424-429.
9. Dinh, K. T.; Sullivan, M. M.; Narsimhan, K.; Serna, P.; Meyer, R. J.; Dincă, M.; Román-Leshkov, Y., Continuous Partial Oxidation of Methane to Methanol Catalyzed by Diffusion-Paired Copper Dimers in Copper-Exchanged Zeolites. *J. Am. Chem. Soc.* **2019,** *141* (29), 11641-11650.
10. Sun, L.; Wang, Y.; Wang, C.; Xie, Z.; Guan, N.; Li, L., Water-Involved Methane-Selective Catalytic Oxidation by Dioxygen Over Copper Zeolites. *Chem* **2021,** *7* (6), 1557-1568.
11. Hirayama, A.; Tsuchimura, Y.; Yoshida, H.; Machida, M.; Nishimura, S.; Kato, K.; Takahashi, K.; Ohyama, J., Catalytic Oxidation of Methane to Methanol over Cu-CHA With Molecular Oxygen. *Catal. Sci. Technol.* **2021,** *11* (18), 6217-6224.
12. Memioglu, O.; Ipek, B., A Potential Catalyst for Continuous Methane Partial Oxidation to Methanol Using N2O: Cu-SSZ-39. *Chem. Commun.* **2021,** *57* (11), 1364-1367.
13. Pokhrel, J.; Shantz, D. F., Continuous Partial Oxidation of Methane to Methanol Over Cu-SSZ-39 Catalysts. *J. Catal.* **2023,** *421*, 300-308.
14. Lyu, Y.; Jocz, J. N.; Xu, R.; Williams, O. C.; Sievers, C., Selective Oxidation of Methane to Methanol over Ceria-Zirconia Supported Mono and Bimetallic Transition Metal Oxide Catalysts. *ChemCatChem* **2021,** *13* (12), 2832-2842.
15. Parfenov, M. V.; Starokon, E. V.; Pirutko, L. V.; Panov, G. I., Quasicatalytic and Catalytic Oxidation of Methane to Methanol by Nitrous Oxide Over FeZSM-5 Zeolite. *Journal of Catalysis* **2014,** *318*, 14-21.
16. Liu, N.; Li, Y.; Dai, C.; Xu, R.; Yu, G.; Wang, N.; Chen, B., H2O In Situ Induced Active Site Structure Dynamics for Efficient Methane Direct Oxidation to Methanol Over Fe-BEA Zeolite. *J. Catal.* **2022,** *414*, 302-312.
17. Martínez-Pérez, M.; De Miguel, R.; Carbonera, C.; Martinez-Julvez, M.; Lostao, A.; Piquer, C.; Gómez-Moreno, C.; Bartolomé, J.; Luis, F., Size-dependent properties of magnetoferritin. *Nanotechnology* **2010,** *21* (46), 465707.

18. Frisch, M. J. T., G. W.; Schlegel, H. B.; Scuseria, G. E.; Robb, M. A.; Cheeseman, J. R.; Scalmani, G.; Barone, V.; Mennucci, B.; Petersson, G. A.; Nakatsuji, H.; Caricato, M.; Li, X.; Hratchian, H. P.; Izmaylov, A. F.; Bloino, J.; Zheng, G.; Sonnenberg, J. L.; Hada, M.; Ehara, M.; Toyota, K.; Fukuda, R.; Hasegawa, J.; Ishida, M.; Nakajima, T.; Honda, Y.; Kitao, O.; Nakai, H.; Vreven, T.; Montgomery, J. A., Jr.; Peralta, J. E.; Ogliaro, F.; Bearpark, M. J.; Heyd, J.; Brothers, E. N.; Kudin, K. N.; Staroverov, V. N.; Kobayashi, R.; Normand, J.; Raghavachari, K.; Rendell, A. P.; Burant, J. C.; Iyengar, S. S.; Tomasi, J.; Cossi, M.; Rega, N.; Millam, N. J.; Klene, M.; Knox, J. E.; Cross, J. B.; Bakken, V.; Adamo, C.; Jaramillo, J.; Gomperts, R.; Stratmann, R. E.; Yazyev, O.; Austin, A. J.; Cammi, R.; Pomelli, C.; Ochterski, J. W.; Martin, R. L.; Morokuma, K.; Zakrzewski, V. G.; Voth, G. A.; Salvador, P.; Dannenberg, J. J.; Dapprich, S.; Daniels, A. D.; Farkas, Ö.; Foresman, J. B.; Ortiz, J. V.; Cioslowski, J.; Fox, D. J. *Gaussian 09, Revision A.02*, Gaussian, Inc.: Wallingford, CT, USA, 2009.
